# Improving the quality of reporting of systematic reviews of dose-response meta-analyses: a cross-sectional survey

**DOI:** 10.1186/s12874-018-0623-6

**Published:** 2018-11-29

**Authors:** Chang Xu, Tong-Zu Liu, Peng-Li Jia, Yu Liu, Ling Li, Liang-Liang Cheng, Xin Sun

**Affiliations:** 1 Chinese Evidence-based Medicine Center and CREAT group, West China Hospital, Sichuan University and Collaborative Innovation Center, 37 Guo Xue Xiang, Chengdu, 610041 China; 2 Department of Urology, Zhongnan Hospital, Wuhan University, Wuhan, China; 3 School of Management, Shanxi Medical University, Taiyuan, China; 4 Gansu Provincial Women and Children Hospital, Gansu, China; 5 West China School of Public Health, Sichuan University, Chengdu, China; 6 Center for Evidence-based Medicine, Jiangxi University of Traditional Chinese Medicine, Nanchang, China

**Keywords:** Dose-response meta-analysis, Reporting quality, Cross-sectional survey

## Abstract

**Background:**

Dose-response meta-analysis (DRMA) is a useful tool to investigate potential dose-response relationship between certain exposure or intervention and the outcome of interest. A large number of DRMAs have been published in the past several years. However, the standard of reporting for such studies is not known.

**Methods:**

Medline, Embase, and Wiley Library were searched for systematic reviews with DRMAs (SR-DRMAs) published from January 2011 to July 2017. We used the combination of PRISMA and MOOSE statements, containing 33 items, to assess the reporting of included SR-DRMAs. The adherence of reporting was defined as the proportion of SR-DRMAs meeting the reporting requirement of an item. We explored the association between five pre-specified variables with the total score of reporting on both fully as well as each domain of the checklist.

**Results:**

In total, 529 SR-DRMAs were eligible. Ten out of 33 items were under reported, and this mainly refers to the methods domain: only a small proportion of SR-DRMAs stated whether a review protocol existed (45, 8.5%); clarified the qualifications of searchers (1.7%); presented full electronic search strategy (25.9%); described any effort to include all available studies (22.9%), described methods for languages other than English (27.4%), and stated the process for selecting studies (20.2%). Multiple regression analysis suggested that studies with more authors (regression coefficient = 0.78; 95% CI: 0.35, 1.20; *P* <  0.001), published more recently (regression coefficient = 0.38; 95% CI: 0.28 to 0.47; trend *P* <  0.001), used reporting guideline (regression coefficient = 0.98; 95% CI: 0.68 to 1.32; *P* <  0.001), and involvement of methodologist (regression coefficient = 0.86; 95% CI: 0.42 to 1.32; *P* <  0.001) were associated with higher score of reporting. Further regression suggested that the improvement on the quality mainly concentrated on the methods and results domains.

**Conclusions:**

The reporting of SR-DRMAs needs to be further improved, particularly in the issues refer to the methods. The quality of reporting may improve when involving more authors and methodologists and employing any reporting guidelines.

**Electronic supplementary material:**

The online version of this article (10.1186/s12874-018-0623-6) contains supplementary material, which is available to authorized users.

## Introduction

Dose-response meta-analysis (DRMA) represents a specific type of meta-analysis that combines, from studies addressing a same question, dose-specific effect estimates of certain exposure on the outcome of interest [1, 2]. One important merit of DRMA is its capacity to explore potentially differential effects according to varying level of exposure [3, 4], which may better inform clinical decisions, particularly when a putative dose-response effect falls into the topic of clinical interest.

An increasing number of systematic reviews (SR) and DRMAs (SR-DRMAs) have been published over the past several years. Clinically meaningful SR-DRMAs largely rely on rigorous design and conduct of study and analysis of data. Nevertheless, the optimal use of findings from such studies also depends on the reporting of SR-DRMAs. Previous studies have highlighted the importance of reporting for systematic review and meta-analysis, and insufficient reporting often results in less effective use of research evidence in clinical practice [5, 6]. In a survey of random sample of systematic reviews, most were found to be poorly reported, particularly for some important aspects of the methods (e.g. literature search, quality assessment) [7]. Lack of such information would reduce effective assessment of quality of evidence.

Up to now, the standard of reporting of published SR-DRMAs is unclear. Although a few studies have been used for the development of clinical practice guideline [8, 9], concerns remain as to whether the reporting information is adequate to support clinical decisions. This is particularly true for DRMAs because of the sophisticated methodologies used in those studies [10]. In order to fully understand the reporting of published SR-DRMAs, we conducted a cross-sectional study to examine the epidemiology of those SR-DRMAs, the quality of reporting and characteristics influencing the reporting.

## Methods

This study was based on a major systematic survey that examined epidemiological characteristics, methodological and reporting quality, and associated characteristics of published SR-DRMAs of exclusively binary outcomes. In this study, we reported the findings about the quality of reporting and associated characteristics.

### Eligibility criteria

We included published SR-DRMAs (aggregate) of binary outcomes across all disease areas. The definition of DRMA has been mentioned in the introduction. The definition of systematic review was based on the Cochrane handbook (version 5.2) [11]. We defined aggregate DRMA as a dose-response meta-analysis that uses aggregate data (study level data). We did not consider pooled analysis as it may failed to employ a comprehensive literature search (at least 1 database). We excluded brief report (i.e. a short demonstration of research results), letter, and conference abstracts since such type of publication contains limited information of reporting items.

### Literature search and screening

We searched MEDLINE, EMBASE, and Wiley online library for SR-DRMAs published between January 2011 and July 2017. No language restriction was applied. We used both MeSH terms and free-text words to develop the search strategy, which was primarily drafted by one experienced author (CX), and finalized after discussion within a group of four investigators with expertise in literature search. The details of search strategy can be found in Additional file 1.

Two methods-trained authors (CX and YL), independently and in duplicate, screened titles and abstracts of searched reports, as well as full texts of potentially eligible articles. Any disagreement was discussed by the two authors; if no consensus was achieved, a third author (XS) would be involve for the final judgment.

### Data collection

Using pre-defined, pilot tested data collection forms, two methods-trained authors (CX and PLJ), independently and in duplicate, extracted data from the eligible articles. They collected the following information from each eligible article: first author’s name, year of publication, journal published in, region of the first author, number of authors listed, affiliations of authors, funding information, databases searched, number of original studies included, primary outcome by disease area, category of subject (i.e. intervention, epidemiology, diagnose, prognosis), reporting checklists employed, type of studies, and polynomial model used for the statistical analysis (e.g. quadratic polynomial, restricted cubic spline).

We used the PRISMA (Preferred Reporting Items for Systematic Reviews and Meta-Analyses) statement [6], with slight modifications, to assess the reporting quality of included SR-DRMAs (CX and YL). We removed the item “*structured summary*”, since the summaries of the studies reporting SR-DRMAs vary considerably across journals, and many journals reporting unstructured summaries often require sufficient details. Second, we slightly modified the wording for three items to make it appropriate for a DRMA. In details, we modified 1) the wording of item *“identify the report as a systematic review, meta-analysis, or both”* into *“identify the report as a systematic review, dose-response meta-analysis, or both”*; 2) the wording of item *“present effect estimates and confidence intervals of each study”* into *“present dose-specific effects and confidence intervals of each study”*; and 3) the wording of item *“present results of each meta–analysis down, with confidence intervals and measures of consistency (best with forest plot)”* into *“present results of each dose-response meta–analysis down, with confidence intervals and measures of consistency (best with pooled dose-response curve)”.* Considering SR-DRMAs were mostly conducted by observational studies that some quality tips may not be covered by PRISMA, we added another 6 items from the MOOSE (Meta-analyses Of Observational Studies in Epidemiology) checklist [12]. These includes *“qualifications of searchers”, “effort to include all available studies”, “use of hand searching”, “method of addressing articles published in languages other than English”, “assessment of confounding”, and “assessment of heterogeneity”*. In addition, the item *“provide a general interpretation of the results in the context of other evidence, and implications for future research”* from PRISMA checklist was presented as two by MOOSE checklist as “*alternative interpretation of the results*” and “*implication for future research*”. We then used the idea from the MOOSE checklist. As a result, the modified checklist included 33 items (Additional file 1: Table S1).

To ensure the assessment of quality, we required that each assessor (CX and YL) should spend at least 30 min for assessing each article, and they were asked to complete up to 20 reports each day. For the two assessors, one (CX) is the co-primary author for the “one-stage” DRMA model of the robust error meta-regression (REMR) [4] and another one (YL) has 3 years’ experience of conducting dose-response meta-analysis.

### Statistical analysis

We summarized the baseline characteristics (e.g. number of authors, year of publish) by descriptive statistics. These include the median value (quartile range) or mean value (standard error) to measure the central tendency and variability for continuous variables, while the count and percentage to measure dichotomous or categorical variables.

For each item, we calculated the adherence rate of each specific reporting items, which was the percentage of all published SR-DRMAs adhering to the item. We judged that an item was well reported if it was reported by 80% or more of the SR-DRMAs, or under reported if less than 80% [13].

We calculated the adherence of published SR-DRMAs to the pre-specified reporting items. We defined that a reporting item was adhered by an individual study if that study reported the information required by the item, and thus assigned one point for that item. If a study met the reporting requirement of all items, a total of 33 points would be assigned for that study (i.e. the total reporting score), which was commonly employed in similar researches [14, 15]. A higher score means better quality.

We pre-specified five variables for exploring their influence on the reporting quality [15, 16]. These were region of first author (Asia Pacific versus European versus America), number of authors (<=4, 5–6, 7–8, and > 8, according to the inter-quartiles), year of publication, involvement of any methodologist (yes versus no), and use of any reporting checklist (yes versus no). We assessed that a SR-DRMA involved a methodologist if any of the authors and those listed in the acknowledgement section was affiliated with department of epidemiology, statistics, mathematics, evidence-based medicine, and public health. The five variables were assessed against multicollinearity. A correlation of less than 0.4 was considered weakly correlated [17].

We used the weighted least square regression to investigate the association between reporting quality and the five pre-specified variables that all variables were entered simultaneously in the regression model [4]. Given the potential correlations of the reporting quality of SR-DRMAs published in the same journal, each journal was treated as a cluster in the regression model. We used generalized estimating equation regression with robust variance as a sensitivity analysis to see if the results were stable. To better understand the effects of the variables on the quality, we further employed a multivariable regression (post hoc) to see the association between pre-specified variables and the quality score of each domain of the checklist (i.e. title and introduction, methods, results, discussion and funding information).

All the analyses were conducted in the Stata14.0/SE software (STATA, College Station, TX, Serial number: 10699393), with alpha = 0.05 as the criterion for statistical significance.

## Results

**Fig. 1:**
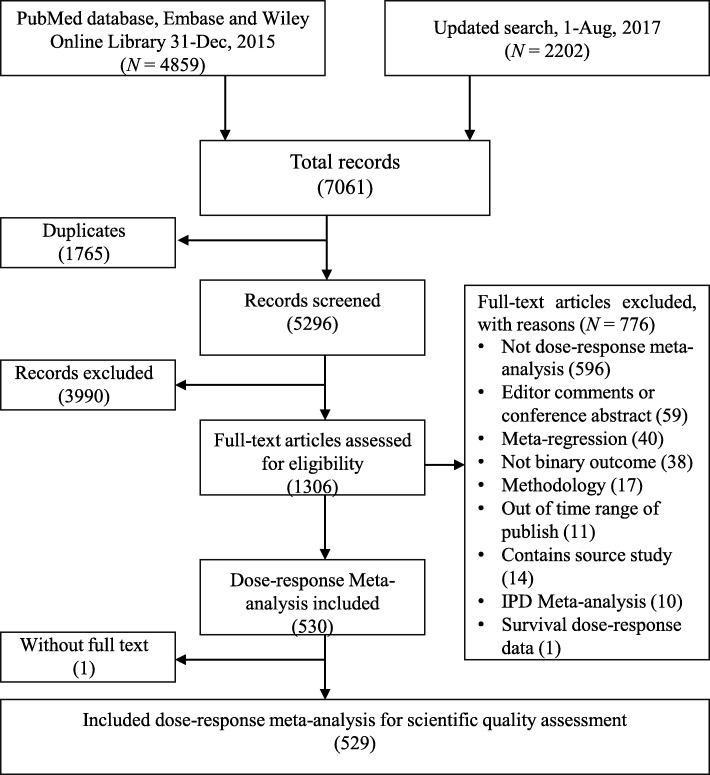
The Flow chart of literature screen

We searched 7061 records. After excluding duplicates and abstract screening, 1306 reports were potentially eligible. By reading full texts, we finally included 529 SR-DRMAs (Fig. 1). A full list of the included SR-DRMAs was presented in supplementary file (Additional file 1: Table S2).

The 529 SR-DRMAs were published in 174 academic journals (number of publications per journal: 1 to 33). Among those, 410 (77.5%) were published in specialist journals and 119 (22.5%) in general journals; 353 (66.8%) were conducted by authors from Asia-Pacific region, 129 (24.4%) from Europe, and 47 (8.9%) from North America. Most of SR-DRMAs (75.0%) were published after 2014. The median number of authors of was 6 [interquartile range (IQR): 4, 8]. The median number of databases searched was 2 (IQR: 2, 3), and 61 studies (11.5%) searched only 1 database. Regarding the reporting guideline, there were 204 (38.6%) of SR-DRMAs used the MOOSE statement, 109 (20.6%) used PRISMA statement, while 179 (33.8%) did not use any of the reporting guidelines. In addition, of those 529 studies, 349 (66.0%) involved methodologist, and 337 (66.0%) received financial supports (Table 1). The majority of the SR-DRMAs focused on epidemiology (*n* = 525, 99.2%). The three most commonly disease outcomes were cancer (*n* = 260, 49.15%), cardiovascular diseases (*n* = 118, 22.31%), and diabetes (*n* = 45, 8.5%).

**Table 1:** Basic characteristics of published DRMA in past 7 years

Category by items	All publications (*N* = 529)
No. of authors [median (first to third quartile)]	6 (4 to 8)
≤ 4	171 (32.33%)
5 ~ 8	278 2.55%)
> 8	80 (15.12%)
Year of publish	
2011	35 (6.62%)
2012	44 (8.32%)
2013	56 (10.59%)
2014	117 (22.12%)
2015	120 (22.68%)
2016	85 (16.07%)
2017 (up to July-31)	72 (13.61%)
Database searched [median ((first to third quartile))]	2 (2 to 3)
≤ 1	61 (11.53%)
2 ~ 3	385 (72.78%)
> 3	83 (15.69%)
Journal distribution (*n* = 174 for journal numbers)	
Specialist journal	410 (77.50%)
General journal	119 (22.50%)
Methodologist involved	
Yes	349 (65.97%)
No	180 (34.03%)
Design of source study	
Cohort	318 (60.11%)
Case-control	7 (1.32%)
Cross-section	3 (0.57%)
Mixed	199 (37.62%)
CCT and RCT	2 (0.38%)
Classification of subject	
Epidemiology	525 (99.24%)
Intervention	1 (0.19%)
Prognosis	2 (0.38%)
Diagnose	1 (0.19%)
Primary outcome	
Cancer	260 (49.15%)
CVD	118 (22.31%)
Type 2 Diabetes	45 (8.51%)
Fracture and Osteoarthritis	21 (3.97%)
CVD and Cancer/Diabetes	13 (2.46%)
Metabolic Syndrome or Obesity	13 (2.46%)
Pregnancy Outcomes (e.g. neonatal death, low birth weight)	12 (2.27%)
Dementia/Cognitive impairment/Alzheimer’s Disease/Parkinson’s disease	12 (2.27%)
Depression	6 (1.13%)
Digestive tract disease (e.g. pancreatitis, gallbladder disease)	12 (2.27%)
Urinary System disease (e.g. urolithiasis)	6 (1.13%)
Others (e.g. Cataract, Gout)	12 (2.27%)
No. of included studies [median ((first to third quartile))]	14 (10 to 21)
≤ 10	151 (28.54%)
11 ~ 21	247 (46.69%)
> 21	130 (24.57%)
Missing	1 (0.19%)
Region	
Asian	350 (66.16%)
European	129 (24.39%)
America	47 (8.88%)
Australia	3 (0.57%)
Reporting checklist	
PRISMA	109 (20.60%)
MOOSE	204 (38.56%)
PRISMA + MOOSE	31 (5.86%)
Other	6 (1.13%)
None	179 (33.84%)
Model used in trend approximation^a^	
RCS regression	295 (55.77%)
FP regression	61 (11.53%)
Other non-linear regression	21 (3.97%)
Linear	152 (28.73%)
Funding	
Yes	337 (63.71%)
No	54 (10.21%)
Not reported	138 (26.09%)

^a^*RCS* restricted cubic spline, *FP* fractional polynomial; other non-linear regression including natural cubic spline, quadratic polynomial, et al.

### Reporting quality of included SR-DRMAs

**Fig. 2:**
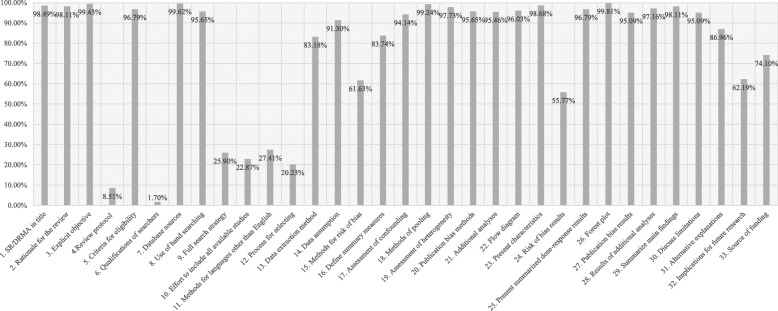
The adherence rate of each reporting items

Figure 2 presents the adherence of published SR-DRMAs to each of the reporting item. The overall score for reporting quality was 25.52 (standard error: 2.36; median 26, first – third quartile: 24, 27). In summary, of those 33 items, 23 were highly adhered to by the SR-DRMAs. Ten items (10/33) were under reported by these SR-DRMAs, while 7 of which refer to the methods domain. The details were as follows.

For the reporting of title and introduction, all of the three items were highly adhered. These included: identified the report as a systematic review (*n* = 521, adherence rate = 98.5%), described the rationale in the introduction (*n* = 519, 98.1%), provided an explicit objective in the introduction (*n* = 526, 99.4%).

For the reporting of methods domain, 11 out of the 18 items were highly adhered to by these SR-DRMAs, while 7 were under reported. Highly adhered items: specified criteria for eligibility (*n* = 512, 96.8%), described database sources (*n* = 527, 99.6%), described the use of hand searching (*n* = 506, 95.7%), described method of data extraction (*n* = 440, 83.2%), described any variable definition and data assumption (*n* = 483, 91.3%), stated the principal summary measures (*n* = 443, 83.7%), stated the methods for confounding assessment (*n* = 498, 94.1%), described methods for combining results (*n* = 525, 99.2%), described methods for heterogeneity assessment (*n* = 517, 97.7%), stated the methods for publication bias (*n* = 506, 95.7%), described methods of additional analyses (*n* = 505, 95.5%). Under reported items: indicated that a review protocol exists (*n* = 45, 8.5%), clarified the qualifications of searchers (*n* = 9, 1.7%), presented full electronic search strategy (*n* = 137, 25.9%), described any (or no) effort to include all available studies (*n* = 121, 22.9%), described methods for languages other than English (*n* = 145, 27.4%), stated the process for selecting studies (*n* = 107, 20.2%), described methods used for assessing risk of bias (*n* = 326, 61.6%).

For the reporting of results domain (7 items), all but 1 were highly adhered. Highly adhered items: presented the screen process and eligible studies (*n* = 508, 96.0%), presented characteristics of included studies (*n* = 522, 98.7%), presented summarized dose-response relationship and confidence interval (*n* = 512, 96.8%), presented results of each study (*n* = 528, 99.8%), presented results of publication bias (*n* = 503, 95.1%), presented results of additional analysis (*n* = 514, 97.2%). Under adhered item: presented results of risk of bias (*n* = 295, 55.8%).

For the reporting of discussion and funding information (5 items), 3 of which were highly adhered while 2 were under reported. Highly adhered items: summarized the main findings (*n* = 519, 98.1%), discussed the general limitations (n = 503, 95.1%), provided general interpretation of the results (*n* = 460, 86.96%). Under adhered items: provided implications for future research (*n* = 329, 62.2%), and described sources of funding (*n* = 392, 74.1%).

### Study characteristics associated with reporting quality

**Fig. 3:**
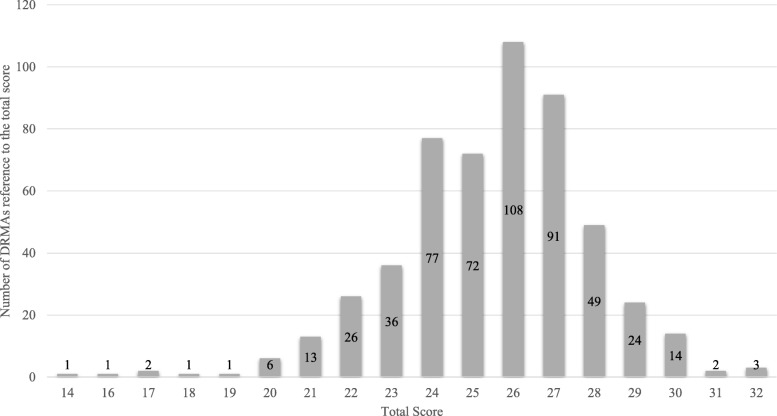
The distribution of global reporting quality

Figure 3 presents the distribution of overall reporting quality scores across those 529 studies. There was no obvious multicollinearity among the five variables. In the multivariable regression analysis, studies with a larger number of authors [5 to 6 vs. 4 or less (regression coefficient = 0.78; 95% CI: 0.35, 1.20; *P* <  0.001)], studies published more recently (regression coefficient = 0.38; 95% CI: 0.28, 0.47; P for trend < 0.001), the use of reporting guideline (regression coefficient = 0.98; 95% CI: 0.63, 1.32), and involvement of methodologist (regression coefficient = 0.86; 95% CI: 0.42, 1.32; *P* <  0.001) were statistically associated with better reporting quality (Table 2). The sensitivity analysis showed that the results were similar (Table 2).

**Table 2:** Multivariable regression analysis of potential factors for reporting quality

Influential factors	Estimated regression coefficients (95%CI)			
	Multivariable	*P-*value	Sensitivity analysis	*P-*value
No. of authors				
≤ 4	Reference		Reference	
5 ~ 6	0.78 (0.35, 1.20)	< 0.001	0.73 (0.28, 1.18)	0.002
7~ 8	0.86 (0.37, 1.36)	0.001	0.68 (0.18, 1.19)	0.008
> 8	1.15 (0.61, 1.70)	< 0.001	0.99 (0.48, 1.49)	< 0.001
Year of publication				
2011	Reference		Reference	
2012	0.39 (−0.41, 1.20)	0.338	0.77 (− 0.05, 1.58)	0.066
2013	1.23 (0.28, 2.18)	0.011	1.12 (0.18, 2.05)	0.020
2014	0.93 (0.13, 1.74)	0.023	1.08 (0.20, 1.96)	0.016
2015	1.35 (0.58, 2.11)	0.001	1.39 (0.54, 2.25)	0.001
2016	2.01 (1.28, 2.75)	< 0.001	2.19 (1.38, 3.00)	< 0.001
2017 (up to July-31)	2.39 (1.60, 3.18)	< 0.001	2.56 (1.62, 3.52)	< 0.001
Linear trend test	0.38 (0.28, 0.47)	< 0.001	0.38 (0.27, 0.50)	< 0.001
Use of reporting guidance				
No	Reference		Reference	
Yes	0.98 (0.63, 1.32)	< 0.001	0.99 (0.61, 1.37)	< 0.001
Region				
European	Reference		Reference	
Asia Pacific	−0.21 (− 0.66, 0.23)	0.348	− 0.28 (− 0.74, 0.17)	0.224
America	− 0.18 (−1.31, 0.95)	0.752	− 0.54 (−1.61, 0.53)	0.320
Methodologist involved				
No	Reference		Reference	
Yes	0.86 (0.42, 1.32)	< 0.001	0.78 (0.36, 1.19)	< 0.001

The multivariable regression was based on weighted least square linear regression; the sensitivity analysis was based on generalized estimating equation (GEE); both the two methods with the variance estimation based on robust standard error

The correlations of the quality score among the four domains were small (range from 0.003 to 0.357). Therefore, the regression for each domain was conducted separately (Additional file 2). The results showed that, number of authors (more authors), year of publication (more recently), the use of reporting guideline, and involvement of methodologist mainly contributed the reporting quality of the methods and results domains while not associated with the reporting of the title and introduction (Table 3).

**Table 3:** Multivariable regression analysis of potential factors for reporting quality of each domain

Influential factors	Reporting domains and estimated regression coefficients							
	Title and Introduction	*P*-value	Methods	*P*-value	Results	*P*-value	Conclusion	*P*-value
No. of authors								
≤ 4	Reference		Reference		Reference		Reference	
5 ~ 6	0.02 (−0.03, 0.06)	0.457	0.50 (0.20, 0.80)	0.001	0.17 (0.03, 0.31)	0.018	0.20 (0.05, 0.36)	0.011
7~ 8	−0.01 (− 0.05, 0.04)	0.819	0.57 (0.22, 0.92)	0.002	0.09 (−0.05, 0.23)	0.208	0.10 (−0.10, 0.30)	0.315
> 8	0.04 (−0.02, 0.09)	0.179	0.64 (0.23, 1.04)	0.002	0.10 (−0.07, 0.27)	0.253	0.33 (0.12, 0.55)	0.003
Year of publication								
2011	Reference		Reference		Reference		Reference	
2012	0.01 (−0.00, 0.02)	0.198	0.37 (−0.30, 1.04)	0.279	0.36 (0.05, 0.68)	0.025	−0.29 (− 0.69, 0.11)	0.157
2013	0.00 (−0.01, 0.01)	0.819	0.68 (−0.04, 1.41)	0.062	0.45 (0.15, 0.75)	0.004	−0.15 (− 0.49, 0.19)	0.387
2014	0.01 (0.00, 0.02)	0.048	0.49 (0.06, 1.33)	0.142	0.54 (0.23, 0.85)	0.001	−0.19 (− 0.55, 0.18)	0.318
2015	0.00 (−0.02, 0.02)	0.964	0.69 (0.06, 1.33)	0.032	0.65 (0.34, 0.96)	< 0.001	−0.04 (− 0.40, 0.31)	0.818
2016	−0.00 (− 0.02, 0.01)	0.709	1.03 (0.41, 1.64)	0.001	0.81 (0.53, 1.08)	< 0.001	0.10 (−0.24, 0.45)	0.552
2017 (up to July-31)	0.00 (−0.02, 0.01)	0.914	1.50 (0.82, 2.18)	< 0.001	0.83 (0.53, 1.13)	< 0.001	−0.16 (− 0.53, 0.21)	0.399
Use of reporting guidance								
No	Reference		Reference		Reference		Reference	
Yes	−0.00 (−0.02, 0.04)	0.083	0.57 (0.32, 0.82)	< 0.001	0.25 (0.13, 0.38)	< 0.001	0.17 (0.03, 0.32)	0.020
Region								
European	Reference		Reference		Reference		Reference	
Asia Pacific	−0.03 (−0.06, − 0.001)	0.042	− 0.45 (− 0.77, − 0.13)	0.006	0.24 (0.07, 0.41)	0.005	−0.10 (− 0.26, 0.06)	0.230
America	0.00 (−0.06, 0.01)	0.637	−0.14 (− 0.93, 0.65)	0.720	−0.08 (− 0.36, 0.19)	0.552	−0.14 (− 0.39, 0.12)	0.302
Methodologist involved								
No	Reference		Reference		Reference		Reference	
Yes	−0.00 (−0.02, 0.02)	0.859	0.32 (0.06, 0.58)	0.015	0.10 (−0.02, 0.22)	0.095	0.40 (0.24, 0.56)	< 0.001

Note: The correlations of the four domains were small, ranges from 0.003 to 0.357

## Discussion

In this study, we found that the adherence in two-thirds of the reporting items of SR-DRMAs was generally good. Reporting on some items, however, needs to be improved, especially those items refer to the methods domain. These including indication of a review protocol, clarifying the qualifications of searchers, statement of any effort to include all available studies, description methods for languages other than English, presentation of full electronic search strategy, statement about the process for selecting studies, description about methods used for assessing risk of bias, presentation of results about risk of bias, presentation of implications for future research, and statement about sources of funding. In particular, the reporting about study protocol and qualifications of searchers are the two least reported items. The under-reporting about study protocol is partly because some journals have not required registration of systematic reviews. The failure to report the qualifications of searchers is probably due to librarians were seldom involved in such types of meta-analysis. In addition, very few authors failed to report whether they taken any effort to get all available studies.

We also found that studies with more authors and involvement of methodologists were associated with better reporting. Our further analysis suggested that this positive effect was mainly due to the improvement of the methods domain. This highlighted the importance of inviting collaborations in the conduct and reporting of systematic reviews. In particular, SR-DRMAs are usually more sophisticated than traditional meta-analyses of pair-wise comparisons. The methodological sophistication often requires more careful planning and reporting of methods details. Involvement of more authors with diverse backgrounds, particularly those who have methodological expertise, would improve the quality of such studies. In the regression analysis, we also found that the use of reporting guideline was associated with better reporting quality, detailed in the methods domain, the results domain, and the conclusion domain. In addition, the trend test within the multiple regression showed that the quality of reporting has improved over the years. Similarly, the improvement of the reporting mainly reflected in the methods and results domains. This finding is supported by a previous survey of meta-analyses of vascular surgery [18]. These represent a good phenomenon in the scientific reporting of SR-DRMAs.

The findings of adherence were of somewhat similar to an earlier comprehensive survey of the reporting of SRs [19]. In that research, the authors found that less than 6% of the SRs provided a protocol, 38% specified the method for risk of bias assessment, and 30% presented the results of risk of bias. The adherence rate of the three items in current study were 8.5, 61.6, and 55.8% respectively. In addition, in both of our survey on SR-DRMAs and the previous survey on all SRs, the title, introduction, eligible criteria, source of database, summery measurements, and limitations were generally well complied.

In a recent survey on meta-analysis, authors reported that financial support was associated with better reporting [20]. Gagnier et al. [21] observed positive association between reporting of funding source and the whole reporting quality. In our study, we did not include the funding information in the regression analysis, mainly because the item “reporting of financial information” was part of the PRISMA statement. Nevertheless, the reporting of financial information still needs to be improved since about a quarter of the SR-DRMAs failed to provide this information, while this issue is more serious (almost 2/3) in the previous survey [19].

There were differences between our findings and earlier studies. One study [20] included all types of meta-analysis in urological literature and categorized reporting quality as binary outcome (superior quality vs. non-superior), but did not observe any association between number of authors and superior quality. Nagendrababu et al. [22] included all types of meta-analysis in Endodontics also found no association between number of authors and reporting quality. Gagnier et al. assessed the focused on orthopaedic systematic reviews, and found no difference of the reporting quality over publication years [21]. Adie et al. [23] summarized the meta-analyses of surgical interventions, but they did not find significant difference of the reporting quality with the involvement of methodologist. In our study, we observed significant association between number of authors, year of publication, involvement of methodologist and reporting quality. One possible explanation is the different “subject” of the three studies. In our study, we only focus on dose-response meta-analysis while the others considered different type of meta-analysis. Another reason may be that DRMA is more sophisticated than the traditional meta-analysis, and researchers involved in this type of meta-analyses may have been more aware of systematic review methodology, including reporting.

Our study has several strengths. To the best of our knowledge, this is the first study that specifically assessed the reporting of dose-response meta-analyses. We included nearly all of the published SR-DRMAs. Thus the findings would be of highly representative. We pre-specified a limited number of variables for exploring association between study characteristics and reporting quality. In addition, we used rigorous analytical approach to address cluster effect in the regression analysis, and conducted sensitivity analyses which showed robustness of findings.

The study has a few limitations. First, the approach we used to measure quality of reporting did not consider relative importance among items (all the items assumed to carry equal weight). Thus, the scoring scheme on reporting may not be optimal. However, there has not been a validated approach to assign weight to each of the item. The current approach may represent the reality one would have face in the exploration of the study characteristics with the reporting. In addition, this approach has been widely used [15, 19–22]. Secondly, our survey excluded SR-DRMAs which involved continuous outcomes. This decision was made mainly because very few SR-DRMAs used a continuous outcome. The finding may thus not be applicable to those SR-DRMAs which reported continuous outcomes and further investigation is warranted. In addition, there was no an existing reporting guideline specific for SR-DRMA, we used the combination of PRISMA and MOOSE statements to assess the reporting quality may be insufficient to map the “real” situations, especially for those DRMAs without systematic review.

## Conclusions

In conclusion, by the current evidence, the reporting of SR-DRMAs on some domains (introduction, results) were generally good, while suboptimal in the methods domain. However, there were the risk that some potential aspects of the reporting for DRMA were not fully covered and requires further investigation. Further efforts are needed to improve the reporting, particularly on several items, such as study protocol and qualifications of searchers. SR-DRMAs involving more authors and methodologists, used of any reporting guideline, and published more recently may be benefited with better reporting quality. It is necessary for methodologists to develop a reporting guideline specific for DRMA.

## Additional files

Additional file 1: Search strategy, modified checklist for quality assessment, and list of included DRMAs. (DOCX 68 kb)

Additional file 2: The raw data we used for the regression analysis. This contains 6 variables (e.g year of publication, journal), the total reporting score in all, and the total score of each domain. (XLSX 40 kb)
