# Supplementary material for: Improving the quality of reporting of systematic reviews of dose-response meta-analyses: a cross-sectional survey
Source: BMC Med Res Methodol. 2018 Nov 29;18:157. doi: 10.1186/s12874-018-0623-6 (PMC6267919; doi:10.1186/s12874-018-0623-6)
Supplement: Supplementary file 1 — Search strategy, modified checklist for quality assessment, and list of included DRMAs. (DOCX 68 kb) [file 12874_2018_623_MOESM1_ESM.docx]

**Search Strategy**

**Ovid MEDLINE(R) In-Process & Other Non-Indexed Citations and Ovid MEDLINE(R) 1946 to Present (2011 to 2015-12-31)**

| **Items** | **Searches** | **Results** | **Search Type** |
| --- | --- | --- | --- |
| 1 | Dose Response Relationship*/exp {No Related Terms} | 473 | Basic |
| 2 | Dose-response association*/exp {No Related Terms} | 9478 | Basic |
| 3 | Dose-effect*/exp {No Related Terms} | 6287 | Basic |
| 4 | or/1-3 | 16162 | Advanced |
| 5 | Meta-analysis/exp {No Related Terms} | 19789 | Basic |
| 6 | ''Pool? Analysis'' {No Related Terms} | 8322 | Basic |
| 7 | Systematic review*/exp {No Related Terms} | 15451 | Basic |
| 8 | or/5-7 | 39299 | Advanced |
| 9 | 4 and 8 | 120 | Advanced |
| 10 | (''dose-response meta-analysis'' or ''non-linear meta-regression'' or ''meta-analysis of prospective studies'' or ''meta-analysis of cohort studies'' or ''meta-analysis of observational studies'').tw,ot. | 1613 | Advanced |
| 11 | 9 or 10 | 1730 | Advanced |
| 12 | animals/ not (Humans/ and Animals/) {No Related Terms} | 10028 | Basic |
| 13 | 11 not 12 | 1730 | Advanced |
| **14** | **13 and 2011:2015.(sa_year).** | **1381** | **Advanced** |

**Embase database**

| **No.** | **Query** | **Results** |
| --- | --- | --- |
| #19 | **#18** AND [embase]/lim | **2,474** |
| #18 | **#17** AND (**'evidence based medicine'**/de OR **'human'**/de OR **'meta analysis'**/de OR **'meta analysis (topic)'**/de OR **'systematic review'**/de) AND (**2011**:py OR **2012**:py OR **2013**:py OR **2014**:py OR **2015**:py) | **2,598** |
| #17 | **#10** OR **#16** | **6,450** |
| #16 | **#11** OR **#12** OR **#13** OR **#14** OR **#15** | **1,832** |
| #15 | **'meta-analysis of observational studies'** | **1,008** |
| #14 | **'meta-analysis of cohort studies'** | **290** |
| #13 | **'meta-analysis of prospective studies'** | **375** |
| #12 | **'non-linear meta-regression'** | **1** |
| #11 | **'dose-response meta-analysis'** | **262** |
| #10 | **#5 AND #9** | **4,788** |
| #9 | **#6 OR #7 OR #8** | **164,355** |
| #8 | **'systematic review'/exp** | **101,893** |
| #7 | **'pool? analysis'** | **17** |
| #6 | **'meta analysis'**/exp | **102,438** |
| #5 | **#1** OR **#2** OR **#3** OR **#4** | **372,838** |
| #4 | **'dose-effects'** | **1,534** |
| #3 | **'dose-effect'**/exp | **371,631** |
| #2 | **'dose-response association'** | **485** |
| #1 | **'dose response relationship'**/exp | **371,631** |

**Wiley Online Library (2011~2015)** *1004 results were presented*

(''dose-response meta-analysis'' or ''non-linear meta-regression'' or ''meta-analysis of prospective studies'' or ''meta-analysis of cohort studies'' or ''meta-analysis of observational studies'') in Abstract NOT animal* in All Fields between years 2011 and 2015

**Table S1**. Modified checklists

| **Modified PRISMA** |
| --- |
| Item 1 (Title): Identify the report as a systematic review, dose-response meta-analysis, or both. |
| Item 2 (Introduction): Describe the rationale for the review in the context of what is already known. |
| Item 3 (Introduction): Provide an explicit objective(s) with reference to PICOS principle. |
| Item 4 (Methods): Indicate if a review protocol exists, if and where it can be accessed (e.g., Web address), and, if available, provide registration information including registration number. |
| Item 5 (Methods): Specify criteria for eligibility, giving rationale. |
| Item 6 (Methods): Specify qualifications of searchers (e.g. librarians and investigators). |
| Item 7 (Methods): Describe all information sources (e.g. databases) in the search and date last searched. |
| Item 8 (Methods): Use of hand searching. |
| Item 9 (Methods): Present full electronic search strategy for at least one database. |
| Item 10 (Methods): Specify any effort to include all available studies, including contact with authors. |
| Item 11 (Methods): Specify method of addressing articles published in languages other than English. |
| Item 12 (Methods): State the process for selecting studies (two stage: title and abstract screen, then the full text). |
| Item 13 (Methods): Describe method of data extraction and any processes for obtaining and confirming data. |
| Item 14 (Methods): List and define all variables for which data were sought and any assumptions made. |
| Item 15 (Methods): Describe methods used for assessing risk of bias of individual studies. |
| Item 16 (Methods): State the principal summary measures (e.g., risk ratio, difference in means). |
| Item 17 (Methods): Specify methods to account for confounding (comparability of cases and controls). |
| Item 18 (Methods): Describe the methods of handling data and combining results of studies. |
| Item 19 (Methods): Specify methods for assessment of heterogeneity (variation in effects). |
| Item 20 (Methods): Specify any assessment of risk of bias for the pooled evidence (e.g. publication bias). |
| Item 21 (Methods): Describe methods of additional analyses (e.g. sensitivity analysis, meta-regression). |
| Item 22 (Results): Give numbers of studies screened, assessed for eligibility, and included in the review, with reasons for exclusions at each stage, ideally with a flow diagram. |
| Item 23 (Results): For each study, present characteristics for which data were extracted. |
| Item 24 (Results): Present data on risk of bias of within each study (study level). |
| Item 25 (Results): Present dose-specific effects and confidence intervals (statistical uncertainty) of each study. |
| Item 26 (Results): Present results of each dose-response meta–analysis down, with confidence intervals and measures of consistency (best with pooled dose-response curve). |
| Item 27 (Results): Present results of risk of bias across studies (publication bias, outcome level). |
| Item 28 (Results): Give results of additional analyses, if done. |
| Item 29 (Discussion): Summarize the main findings including the strength of evidence for each main outcome. |
| Item 30 (Discussion): Discuss limitations at study and outcome level |
| Item 31 (Discussion): Provide a general interpretation of the results. |
| Item 32 (Discussion): Consider implications for future research (Just clarify further study is needed to verify the results or further work on the mechanism are insufficient). |
| Item 33 (Funding): Describe sources of funding and other support for the systematic review. |

**Table S2. Full list of included DRMAs (n=529).**

| **First Author** | **Title** | **Citation** |
| --- | --- | --- |
| Abar L | Blood concentrations of carotenoids and retinol and lung cancer risk: an update of the WCRF-AICR systematic review of published prospective studies. | Cancer Med. 2016 Aug;5(8):2069-83. |
| Ai M | Safety and efficacy of cell-based therapy on critical limb ischemia: A meta-analysis. | Cytotherapy. 2016 Jun;18(6):712-24. |
| Aune D | Nut consumption and risk of cardiovascular disease, total cancer, all-cause and cause-specific mortality: a systematic review and dose-response meta-analysis of prospective studies. | BMC Med. 2016 Dec 5;14(1):207. |
| Aune D | Whole grain consumption and risk of cardiovascular disease, cancer, and all cause and cause specific mortality: systematic review and dose-response meta-analysis of prospective studies. | BMJ. 2016 Jun 14;353:i2716. |
| Aune D | Physical activity and the risk of gestational diabetes mellitus: a systematic review and dose-response meta-analysis of epidemiological studies. | Eur J Epidemiol. 2016 Oct;31(10):967-997. |
| Aune D | Tobacco smoking and the risk of diverticular disease - a systematic review and meta-analysis of prospective studies. | Colorectal Dis. 2017 Jul;19(7):621-633. |
| Aune D | Body Mass Index, Abdominal Fatness, and Heart Failure Incidence and Mortality: A Systematic Review and Dose-Response Meta-Analysis of Prospective Studies. | Circulation. 2016 Feb 16;133(7):639-49. |
| Aune D | Resting heart rate and the risk of cardiovascular disease, total cancer, and all-cause mortality e A systematic review and doseeresponse meta-analysis of prospective studies | Nutrition, Metabolism & Cardiovascular Diseases. 2017, 27, 504e517 |
| Aune D | BMI and all cause mortality: systematic review and non-linear dose-response meta-analysis of 230 cohort studies with 3.74 million deaths among 30.3 million participants. | BMJ. 2016 May 4;353:i2156. |
| Aune D | Body mass index, abdominal fatness, fat mass and the risk of atrial fibrillation: a systematic review and dose-response meta-analysis of prospective studies. | Eur J Epidemiol. 2017 Mar;32(3):181-192. |
| Aune D | Tobacco smoking and the risk of gallbladder disease. | Eur J Epidemiol. 2016 Jul;31(7):643-53. |
| Bai Y | Parity and bladder cancer risk: a dose-response meta-analysis. | BMC Cancer. 2017 Jan 6;17(1):31. |
| Bo Y | Association between dietary vitamin C intake and risk of esophageal cancer: A dose-response meta-analysis. | Int J Cancer. 2016 Apr 15;138(8):1843-50. |
| Boyd RA | Direct Oral Anticoagulants Vs. Enoxaparin for Prevention of Venous Thromboembolism Following Orthopedic Surgery: A Dose-Response Meta-analysis. | Clin Transl Sci. 2017 Jul;10(4):260-270. |
| Chen C | Association between omega-3 fatty acids consumption and the risk of type 2 diabetes: A meta-analysis of cohort studies. | J Diabetes Investig. 2017 Jul;8(4):480-488. |
| Chen F | Carotenoid intake and risk of non-Hodgkin lymphoma: a systematic review and dose-response meta-analysis of observational studies. | Ann Hematol. 2017 Jun;96(6):957-965. |
| Chen GC | Central obesity and risks of pre- and postmenopausal breast cancer: a dose-response meta-analysis of prospective studies. | Obes Rev. 2016 Nov;17(11):1167-1177. |
| Chen GC | Cheese consumption and risk of cardiovascular disease: a meta-analysis of prospective studies. | Eur J Nutr. 2017 Dec;56(8):2565-2575. |
| Chen GC | Whole-grain intake and total, cardiovascular, and cancer mortality: a systematic review and meta-analysis of prospective studies. | Am J Clin Nutr. 2016 Jul;104(1):164-72. |
| Chen J | Parity and gastric cancer risk: a systematic review and dose-response meta-analysis of prospective cohort studies. | Sci Rep. 2016 Jan 4;6:18766. |
| Chen LW | Maternal caffeine intake during pregnancy and risk of pregnancy loss: a categorical and dose-response meta-analysis of prospective studies. | Public Health Nutr. 2016 May;19(7):1233-44. |
| Chen Q | Adult weight gain and risk of prostate cancer: A dose-response meta-analysis of observational studies. | Int J Cancer. 2016 Feb 15;138(4):866-74. |
| Chen S | Dietary fibre intake and risk of breast cancer: A systematic review and meta-analysis of epidemiological studies. | Oncotarget. 2016 Dec 6;7(49):80980-80989. |
| Chen X | The contribution of serum hepatitis B virus load in the carcinogenesis and prognosis of hepatocellular carcinoma: evidence from two meta-analyses. | Oncotarget. 2016 Aug 2;7(31):49299-49309. |
| Chen Y | An inverse association between tea consumption and colorectal cancer risk. | Oncotarget. 2017 Jun 6;8(23):37367-37376. |
| de Goede | Dairy Consumption and Risk of Stroke: A Systematic Review and Updated Dose-Response Meta-Analysis of Prospective Cohort Studies. | J Am Heart Assoc. 2016 May 20;5(5). pii: e002787. |
| Fang X | Dose-Response Relationship between Dietary Magnesium Intake and Risk of Type 2 Diabetes Mellitus: A Systematic Review and Meta-Regression Analysis of Prospective Cohort Studies. | Nutrients. 2016 Nov 19;8(11). pii: E739. |
| Fang X | Dose-response relationship between dietary magnesium intake and cardiovascular mortality: A systematic review and dose-based meta-regression analysis of prospective studies. | J Trace Elem Med Biol. 2016 Dec;38:64-73. |
| Fang X | Dietary magnesium intake and the risk of cardiovascular disease, type 2 diabetes, and all-cause mortality: a dose-response meta-analysis of prospective cohort studies. | BMC Med. 2016 Dec 8;14(1):210. |
| Gijsbers L | Consumption of dairy foods and diabetes incidence: a dose-response meta-analysis of observational studies. | Am J Clin Nutr. 2016 Apr;103(4):1111-24. |
| Gong F | Chocolate Consumption and Risk of Heart Failure: A Meta-Analysis of Prospective Studies. | Nutrients. 2017 Apr 20;9(4). pii: E402. |
| Gong TT | Cholesterol consumption and risk of endometrial cancer: a systematic review and dose-response meta-analysis of observational studies. | Oncotarget. 2016 Mar 29;7(13):16996-7008. |
| Goto E | Dose-response association between maternal body mass index and small for gestational age: a meta-analysis. | J Matern Fetal Neonatal Med. 2017 Jan;30(2):213-218. |
| Grosso G | Coffee, tea, caffeine and risk of depression: A systematic review and dose-response meta-analysis of observational studies. | Mol Nutr Food Res. 2016 Jan;60(1):223-34. |
| Grosso G | Dietary Flavonoid and Lignan Intake and Mortality in Prospective Cohort Studies: Systematic Review and Dose-Response Meta-Analysis. | Am J Epidemiol. 2017 Jun 15;185(12):1304-1316. |
| Grosso G | Coffee consumption and risk of all-cause, cardiovascular, and cancer mortality in smokers and non-smokers: a dose-response meta-analysis. | Eur J Epidemiol. 2016 Dec;31(12):1191-1205. |
| Grosso G | Dietary n-3 PUFA, fish consumption and depression: A systematic review and meta-analysis of observational studies. | J Affect Disord. 2016 Nov 15;205:269-281. |
| Gu WJ | Relationship between Annualized Case Volume and Mortality in Sepsis: A Dose-Response Meta-analysis. | Anesthesiology. 2016 Jul;125(1):168-79. |
| Guo J | Milk and dairy consumption and risk of cardiovascular diseases and all-cause mortality: dose-response meta-analysis of prospective cohort studies. | Eur J Epidemiol. 2017 Apr;32(4):269-287. |
| Guo P | Number of parity and the risk of non-Hodgkin lymphomas: a dose-response meta-analysis of observational studies. | Hematology. 2017 Jun;22(5):274-285. |
| Guo P | Number of parity and the risk of gallbladder cancer: a systematic review and dose-response meta-analysis of observational studies. | Arch Gynecol Obstet. 2016 May;293(5):1087-96. |
| Guo P | Higher parity is associated with increased risk of Type 2 diabetes mellitus in women: A linear dose-response meta-analysis of cohort studies. | J Diabetes Complications. 2017 Jan;31(1):58-66. |
| Guo Y | Body mass index and mortality in chronic obstructive pulmonary disease: A dose-response meta-analysis. | Medicine (Baltimore). 2016 Jul;95(28):e4225. |
| Guo Y | Green tea and the risk of prostate cancer: A systematic review and meta-analysis. | Medicine (Baltimore). 2017 Mar;96(13):e6426. |
| Han H | Dose-response relationship between dietary magnesium intake, serum magnesium concentration and risk of hypertension: a systematic review and meta-analysis of prospective cohort studies. | Nutr J. 2017 May 5;16(1):26. |
| Han H | Blood glucose concentration and risk of liver cancer: systematic review and meta-analysis of prospective studies. | Oncotarget. 2017 Jul 25;8(30):50164-50173. |
| He Q | Sleep duration and risk of stroke: a dose-response meta-analysis of prospective cohort studies. | Sleep Med. 2017 Apr;32:66-74. |
| Heslehurst N | Maternal body mass index and post-term birth: a systematic review and meta-analysis. | Obes Rev. 2017 Mar;18(3):293-308. |
| Hidayat K | Calcium intake and breast cancer risk: meta-analysis of prospective cohort studies. | Br J Nutr. 2016 Jul;116(1):158-66. |
| Huang HY | Maternal obesity and the risk of neural tube defects in offspring: A meta-analysis. | Obes Res Clin Pract. 2017 Mar - Apr;11(2):188-197. |
| Huang J | Specific types of alcoholic beverage consumption and risk of type 2 diabetes: A systematic review and meta-analysis. | J Diabetes Investig. 2017 Jan;8(1):56-68. |
| Huang XZ | Aspirin and non-steroidal anti-inflammatory drugs use reduce gastric cancer risk: A dose-response meta-analysis. | Oncotarget. 2017 Jan 17;8(3):4781-4795. |
| Jayedi A | Vitamin D status and all-cause mortality in patients with chronic kidney disease: A systematic review and dose-response meta-analysis. | J Clin Endocrinol Metab 102: 2136–2145, 2017 |
| Jia K | Vitamin B6 Intake and the Risk of Colorectal Cancer: A Meta-Analysis of Prospective Cohort Studies. | Nutr Cancer. 2017 Jul;69(5):723-731. |
| Jiang R | Phyto-oestrogens and colorectal cancer risk: a systematic review and dose-response meta-analysis of observational studies. | Br J Nutr. 2016 Dec;116(12):2115-2128. |
| Jiang WB | Does body mass index correlate with the mortality of prostate cancer? A dose-response meta-analysis of cohort studies | Int J Clin Exp Med 2017;10(1):88-96 |
| Jiang X | Increased Consumption of Fruit and Vegetables Is Related to a Reduced Risk of Cognitive Impairment and Dementia: Meta-Analysis. | Front Aging Neurosci. 2017 Feb 7;9:18. |
| Kennedy OJ | Coffee, including caffeinated and decaffeinated coffee, and the risk of hepatocellular carcinoma: a systematic review and dose-response meta-analysis. | BMJ Open. 2017 May 9;7(5):e013739. |
| Kwon Y | Body Mass Index-Related Mortality in Patients with Type 2 Diabetes and Heterogeneity in Obesity Paradox Studies: A Dose-Response Meta-Analysis. | PLoS One. 2017 Jan 3;12(1):e0168247. |
| Kyu HH | Physical activity and risk of breast cancer, colon cancer, diabetes, ischemic heart disease, and ischemic stroke events: systematic review and dose-response meta-analysis for the Global Burden of Disease Study 2013. | BMJ. 2016 Aug 9;354:i3857. |
| Li B | Consumption of whole grains in relation to mortality from all causes, cardiovascular disease, and diabetes: Dose-response meta-analysis of prospective cohort studies. | Medicine (Baltimore). 2016 Aug;95(33):e4229. |
| Li BL | Dairy consumption and risk of esophageal squamous cell carcinoma: A meta-analysis of observational studies. | Asia Pac J Clin Oncol. 2016 Jun;12(2):e269-79. |
| Li C | Systematic review and meta-analysis suggest that dietary cholesterol intake increases risk of breast cancer. | Nutr Res. 2016 Jul;36(7):627-35. |
| Li F | Red Meat and Processed Meat Consumption and Nasopharyngeal Carcinoma Risk: A Dose-response Meta-analysis of Observational Studies. | Nutr Cancer. 2016 Aug-Sep;68(6):1034-43. |
| Li J | Legume intake and risk of prostate cancer: a meta-analysis of prospective cohort studies. | Oncotarget. 2017 Jul 4;8(27):44776-44784. |
| Li M | Association between fber intake and ischemic stroke risk: a meta-analysis of prospective studies | Int J Clin Exp Med 2017;10(3):4659-4668 |
| Li M | Hyperuricemia and the risk for coronary heart disease morbidity and mortality a systematic review and dose-response meta-analysis. | Sci Rep. 2016 Jan 27;6:19520. |
| Li P | MECHANISMS IN ENDOCRINOLOGY: Parity and risk of type 2 diabetes: a systematic review and dose-response meta-analysis. | Eur J Endocrinol. 2016 Nov;175(5):R231-45. |
| Li T | The dose-response effect of physical activity on cancer mortality: findings from 71 prospective cohort studies. | Br J Sports Med. 2016 Mar;50(6):339-45. |
| Li W | Sleep duration and risk of stroke events and stroke mortality: A systematic review and meta-analysis of prospective cohort studies. | Int J Cardiol. 2016 Nov 15;223:870-876. |
| Li X | Abdominal obesity and risk of hip fracture: a meta-analysis of prospective studies. | Osteoporos Int. 2017 Oct;28(10):2747-2757. |
| Li XH | Association between alcohol consumption and the risk of incident type 2 diabetes: a systematic review and dose-response meta-analysis. | Am J Clin Nutr. 2016 Mar;103(3):818-29. |
| Li Y | Association between physical activity and all cancer mortality: Dose-response meta-analysis of cohort studies. | Int J Cancer. 2016 Feb 15;138(4):818-32. |
| Li Z | Association between pioglitazone use and the risk of bladder cancer among subjects with diabetes mellitus: a dose-response meta-analysis . | Int J Clin Pharmacol Ther. 2017 Mar;55(3):210-219. |
| Liu JP | CORRELATION BETWEEN ALCOHOL CONSUMPTION AND MYOCARDIAL INFARCTION: DOSE-RESPONSE META-ANALYSIS OF 18 COHORT STUDIES | FARMACIA, 2017, Vol. 65, 1 |
| Liu L | Leisure time physical activity and cancer risk: evaluation of the WHO's recommendation based on 126 high-quality epidemiological studies. | Br J Sports Med. 2016 Mar;50(6):372-8. |
| Liu M | Dose-response association of screen time-based sedentary behaviour in children and adolescents and depression: a meta-analysis of observational studies. | Br J Sports Med. 2016 Oct;50(20):1252-1258. |
| Liu QP | Habitual coffee consumption and risk of cognitive decline/dementia: A systematic review and meta-analysis of prospective cohort studies. | Nutrition. 2016 Jun;32(6):628-36. |
| Liu SY | Alcohol intake and Helicobacter pylori infection: a dose-response meta-analysis of observational studies. | Infect Dis (Lond). 2016 Apr;48(4):303-309. |
| Liu TZ | Sleep duration and risk of all-cause mortality: A flexible, non-linear, meta-regression of 40 prospective cohort studies. | Sleep Med Rev. 2017 Apr;32:28-36. |
| Liu X | Association between tea consumption and risk of cognitive disorders: A dose-response meta-analysis of observational studies. | Oncotarget. 2017 Jun 27;8(26):43306-43321. |
| Liu X | Resting heart rate and risk of metabolic syndrome in adults: a dose-response meta-analysis of observational studies. | Acta Diabetol. 2017 Mar;54(3):223-235. |
| Liu X | Dose-Response Association Between Physical Activity and Incident Hypertension: A Systematic Review and Meta-Analysis of Cohort Studies. | Hypertension. 2017 May;69(5):813-820. |
| Lamm SH | A Systematic Review and Meta-Regression Analysis of Lung Cancer Risk and Inorganic Arsenic in Drinking Water. | Int J Environ Res Public Health. 2015 Dec 7;12(12):15498-515. |
| Ornello R | Migraine and body mass index categories: a systematic review and meta-analysis of observational studies. | J Headache Pain. 2015 Mar 28;16:27. |
| Cheng P | Dietary intake of iron, zinc, copper, and risk of Parkinson's disease: a meta-analysis. | Neurol Sci. 2015 Dec;36(12):2269-75. |
| Thomopoulos TP | Maternal and childhood consumption of coffee, tea and cola beverages in association with childhood leukemia: a meta-analysis. | Cancer Epidemiol. 2015 Dec;39(6):1047-59. |
| Macacu A | Active and passive smoking and risk of breast cancer: a meta-analysis. | Breast Cancer Res Treat. 2015 Nov;154(2):213-24. |
| Rao D | Does night-shift work increase the risk of prostate cancer? a systematic review and meta-analysis. | Onco Targets Ther. 2015 Oct 5;8:2817-26. |
| Ben Q | Association Between Consumption of Fruits and Vegetables and Risk of Colorectal Adenoma: A PRISMA-Compliant Meta-Analysis of Observational Studies. | Medicine (Baltimore). 2015 Oct;94(42):e1599. |
| Samokhvalov AV | Alcohol Consumption as a Risk Factor for Acute and Chronic Pancreatitis: A Systematic Review and a Series of Meta-analyses. | EBioMedicine. 2015 Nov 14;2(12):1996-2002. |
| Li Y | Association between resting heart rate and cardiovascular mortality: evidence from a meta-analysis of prospective studies. | Int J Clin Exp Med. 2015 Sep 15;8(9):15329-39. |
| Han J | Dietary Fat Intake and Risk of Gastric Cancer: A Meta-Analysis of Observational Studies. | PLoS One. 2015 Sep 24;10(9):e0138580. |
| Wang Y | Effect of Carotene and Lycopene on the Risk of Prostate Cancer: A Systematic Review and Dose-Response Meta-Analysis of Observational Studies. | PLoS One. 2015 Sep 15;10(9):e0137427. |
| Aune D | Body mass index, abdominal fatness and the risk of gallbladder disease. | Eur J Epidemiol. 2015 Sep;30(9):1009-19. |
| Chen GC | Circulating 25-hydroxyvitamin D and risk of lung cancer: a dose-response meta-analysis. | Cancer Causes Control. 2015 Dec;26(12):1719-28 |
| Rhee J | Maternal Caffeine Consumption during Pregnancy and Risk of Low Birth Weight: A Dose-Response Meta-Analysis of Observational Studies. | PLoS One. 2015 Jul 20;10(7):e0132334. |
| Xu C | Fat Intake Is Not Linked to Prostate Cancer: A Systematic Review and Dose-Response Meta-Analysis. | PLoS One. 2015 Jul 17;10(7):e0131747. |
| Fang X | Landscape of dietary factors associated with risk of gastric cancer: A systematic review and dose-response meta-analysis of prospective cohort studies. | Eur J Cancer. 2015 Dec;51(18):2820-32. |
| Aune D | Physical activity and the risk of type 2 diabetes: a systematic review and dose-response meta-analysis. | Eur J Epidemiol. 2015 Jul;30(7):529-42 |
| Yan A | Does tea consumption correlate to risk of fracture? A meta-analysis. | Int J Clin Exp Med. 2015 Jun 15;8(6):8347-57. |
| Wang Y | Body mass index and risk of primary liver cancer: a meta-analysis of prospective studies. | Oncologist. 2012;17(11):1461-8. |
| Guo J | Red and processed meat intake and risk of breast cancer: a meta-analysis of prospective studies. | Breast Cancer Res Treat. 2015 May;151(1):191-8. |
| Qin B | Body mass index and the risk of rheumatoid arthritis: a systematic review and dose-response meta-analysis. | Arthritis Res Ther. 2015 Mar 29;17:86. |
| Sun JW | Obesity and risk of bladder cancer: a dose-response meta-analysis of 15 cohort studies. | PLoS One. 2015 Mar 24;10(3):e0119313. |
| Gan Y | Consumption of fruit and vegetable and risk of coronary heart disease: a meta-analysis of prospective cohort studies. | Int J Cardiol. 2015 Mar 15;183:129-37. |
| Zhang Z | Nut consumption and risk of stroke. | Eur J Epidemiol. 2015 Mar;30(3):189-96. |
| Huang RX | Fish intake and risk of liver cancer: a meta-analysis. | PLoS One. 2015 Jan 23;10(1):e0096102. |
| Jiang W | Dietary flavonoids intake and the risk of coronary heart disease: a dose-response meta-analysis of 15 prospective studies. | Thromb Res. 2015 Mar;135(3):459-63. |
| Xu C | Self-Fluid Management in Prevention of Kidney Stones: A PRISMA-Compliant Systematic Review and Dose-Response Meta-Analysis of Observational Studies. | Medicine (Baltimore). 2015 Jul;94(27):e1042. |
| Liao Q | The role of circulating adiponectin in prostate cancer: a meta-analysis. | Int J Biol Markers. 2015 Feb 24;30(1):e22-31. |
| Zeng SB | Long-Term Coffee Consumption and Risk of Gastric Cancer: A PRISMA-Compliant Dose-Response Meta-Analysis of Prospective Cohort Studies. | Medicine (Baltimore). 2015 Sep;94(38):e1640. |
| Asemi Z | Total, dietary, and supplemental calcium intake and mortality from all-causes, cardiovascular disease, and cancer: A meta-analysis of observational studies. | Nutr Metab Cardiovasc Dis. 2015 Jul;25(7):623-34. |
| Wu S | Omega-3 fatty acids intake and risks of dementia and Alzheimer's disease: a meta-analysis. | Neurosci Biobehav Rev. 2015 Jan;48:1-9. |
| Jayalath VH | Sugar-sweetened beverage consumption and incident hypertension: a systematic review and meta-analysis of prospective cohorts. | Am J Clin Nutr. 2015 Oct;102(4):914-21. |
| Quansah R | Association of arsenic with adverse pregnancy outcomes/infant mortality: a systematic review and meta-analysis. | Environ Health Perspect. 2015 May;123(5):412-21. |
| Yao X | Dyslipidemia and colorectal cancer risk: a meta-analysis of prospective studies. | Cancer Causes Control. 2015 Feb;26(2):257-268. |
| Keum N | Egg intake and cancers of the breast, ovary and prostate: a dose-response meta-analysis of prospective observational studies. | Br J Nutr. 2015 Oct 14;114(7):1099-107. |
| Bagnardi V | Alcohol consumption and site-specific cancer risk: a comprehensive dose-response meta-analysis. | Br J Cancer. 2015 Feb 3;112(3):580-93. |
| Grosso G | Nut consumption on all-cause, cardiovascular, and cancer mortality risk: a systematic review and meta-analysis of epidemiologic studies. | Am J Clin Nutr. 2015 Apr;101(4):783-93. |
| Liu H | Coffee consumption and prostate cancer risk: a meta-analysis of cohort studies. | Nutr Cancer. 2015;67(3):392-400. |
| Sun X | Meta-analysis: Tobacco smoking may enhance the risk of acute pancreatitis. | Pancreatology. 2015 May-Jun;15(3):286-94. |
| Keum N | Calcium intake and colorectal adenoma risk: dose-response meta-analysis of prospective observational studies. | Int J Cancer. 2015 Apr 1;136(7):1680-7. |
| Kunutsor SK | Gamma glutamyltransferase and metabolic syndrome risk: a systematic review and dose-response meta-analysis. | Int J Clin Pract. 2015 Jan;69(1):136-44. |
| Vieira AR | Fruits, vegetables, and bladder cancer risk: a systematic review and meta-analysis. | Cancer Med. 2015 Jan;4(1):136-46. |
| Larsson SC | Urinary cadmium concentration and risk of breast cancer: a systematic review and dose-response meta-analysis. | Am J Epidemiol. 2015 Sep 1;182(5):375-80. |
| Schlesinger S | Body weight gain and risk of colorectal cancer: a systematic review and meta-analysis of observational studies. | Obes Rev. 2015 Jul;16(7):607-19. |
| Bagheri M | A dose-response meta-analysis of the impact of body mass index on stroke and all-cause mortality in stroke patients: a paradox within a paradox. | Obes Rev. 2015 May;16(5):416-23. |
| Keum N | Visceral adiposity and colorectal adenomas: dose-response meta-analysis of observational studies. | Ann Oncol. 2015 Jun;26(6):1101-9. |
| Duan P | Nonlinear dose-response relationship between radon exposure and the risk of lung cancer: evidence from a meta-analysis of published observational studies. | Eur J Cancer Prev. 2015 Jul;24(4):267-77. |
| Bai XY | Association between Dietary Vitamin C Intake and Risk of Prostate Cancer: A Meta-analysis Involving 103,658 Subjects. | J Cancer. 2015 Jul 28;6(9):913-21. |
| Yang Y | Association between dietary fiber and lower risk of all-cause mortality: a meta-analysis of cohort studies. | Am J Epidemiol. 2015 Jan 15;181(2):83-91. |
| Lin T | Association between adiponectin levels and endometrial carcinoma risk: evidence from a dose-response meta-analysis. | BMJ Open. 2015 Sep 3;5(9):e008541. |
| Ben Q | Alcohol drinking and the risk of colorectal adenoma: a dose-response meta-analysis. | Eur J Cancer Prev. 2015 Jul;24(4):286-95. |
| Aune D | Dairy products, calcium, and prostate cancer risk: a systematic review and meta-analysis of cohort studies. | Am J Clin Nutr. 2015 Jan;101(1):87-117. |
| Peng HY | Elevated homocysteine levels and risk of cardiovascular and all-cause mortality: a meta-analysis of prospective studies. | J Zhejiang Univ Sci B. 2015 Jan;16(1):78-86. |
| Larsson SC | Alcohol consumption and risk of heart failure: a dose-response meta-analysis of prospective studies. | Eur J Heart Fail. 2015 Apr;17(4):367-73. |
| Wang Q | Association of circulating insulin-like growth factor 1 and insulin-like growth factor binding protein 3 with the risk of ovarian cancer: A systematic review and meta-analysis. | Mol Clin Oncol. 2015 May;3(3):623-628. |
| Wu L | Linear reduction in thyroid cancer risk by oral contraceptive use: a dose-response meta-analysis of prospective cohort studies. | Hum Reprod. 2015 Sep;30(9):2234-40. |
| Zhang C | Consumption of beer and colorectal cancer incidence: a meta-analysis of observational studies. | Cancer Causes Control. 2015 Apr;26(4):549-60. |
| Liu XM | Overweight, obesity and risk of all-cause and cardiovascular mortality in patients with type 2 diabetes mellitus: a dose-response meta-analysis of prospective cohort studies. | Eur J Epidemiol. 2015 Jan;30(1):35-45. |
| Touvier M | Cholesterol and breast cancer risk: a systematic review and meta-analysis of prospective studies. | Br J Nutr. 2015 Aug 14;114(3):347-57. |
| Wang Y | Fruit and vegetable consumption and risk of lung cancer: a dose-response meta-analysis of prospective cohort studies. | Lung Cancer. 2015 May;88(2):124-30. |
| Wang X | Systematic review and meta-analysis of the effect of alcohol intake on the risk of urolithiasis including dose-response relationship. | Urol Int. 2015;94(2):194-204. |
| An N | Oral Contraceptives Use and Liver Cancer Risk: A Dose-Response Meta-Analysis of Observational Studies. | Medicine (Baltimore). 2015 Oct;94(43):e1619. |
| Xu X | Does beer, wine or liquor consumption correlate with the risk of renal cell carcinoma? A dose-response meta-analysis of prospective cohort studies. | Oncotarget. 2015 May 30;6(15):13347-58. |
| Zhang YF | Tea consumption and the incidence of cancer: a systematic review and meta-analysis of prospective observational studies. | Eur J Cancer Prev. 2015 Jul;24(4):353-62. |
| Zhou ZY | HbA1c and Lower Extremity Amputation Risk in Patients With Diabetes: A Meta-Analysis. | Int J Low Extrem Wounds. 2015 Jun;14(2):168-77. |
| Wu Y | Fruit and vegetable consumption and risk of type 2 diabetes mellitus: a dose-response meta-analysis of prospective cohort studies. | Nutr Metab Cardiovasc Dis. 2015 Feb;25(2):140-7. |
| Dong X | Tea consumption and the risk of depression: a meta-analysis of observational studies. | Aust N Z J Psychiatry. 2015 Apr;49(4):334-45. |
| Wang A | Macronutrients intake and risk of Parkinson's disease: A meta-analysis. | Geriatr Gerontol Int. 2015 May;15(5):606-16. |
| Xu Q | Association Between Alcohol Consumption and the Risk of Barrett's Esophagus: A Meta-Analysis of Observational Studies. | Medicine (Baltimore). 2015 Aug;94(32):e1244. |
| Da J | Serum Phosphorus and Progression of CKD and Mortality: A Meta-analysis of Cohort Studies. | Am J Kidney Dis. 2015 Aug;66(2):258-65. |
| Pandey A | Dose-Response Relationship Between Physical Activity and Risk of Heart Failure: A Meta-Analysis. | Circulation. 2015 Nov 10;132(19):1786-94. |
| Zhang YP | Systematic review with meta-analysis: coffee consumption and the risk of gallstone disease. | Aliment Pharmacol Ther. 2015 Sep;42(6):637-48. |
| Karahalios A | Weight change and risk of colorectal cancer: a systematic review and meta-analysis. | Am J Epidemiol. 2015 Jun 1;181(11):832-45. |
| Jing Z | Association between height and thyroid cancer risk: a meta-analysis of prospective cohort studies. | Int J Cancer. 2015 Sep 15;137(6):1484-90. |
| Xi B | Sugar-sweetened beverages and risk of hypertension and CVD: a dose-response meta-analysis. | Br J Nutr. 2015 Mar 14;113(5):709-17. |
| Liu H | Fruit and vegetable consumption and risk of bladder cancer: an updated meta-analysis of observational studies. | Eur J Cancer Prev. 2015 Nov;24(6):508-16. |
| Wu QJ | Dietary fatty acids intake and endometrial cancer risk: a dose-response meta-analysis of epidemiological studies. | Oncotarget. 2015 Nov 3;6(34):36081-97. |
| Tang J | Tea consumption and mortality of all cancers, CVD and all causes: a meta-analysis of eighteen prospective cohort studies. | Br J Nutr. 2015 Sep 14;114(5):673-83. |
| Zeng F | Adiponectin and Endometrial Cancer: A Systematic Review and Meta-Analysis. | Cell Physiol Biochem. 2015;36(4):1670-8. |
| Fang X | Dietary intake of heme iron and risk of cardiovascular disease: a dose-response meta-analysis of prospective cohort studies. | Nutr Metab Cardiovasc Dis. 2015 Jan;25(1):24-35. |
| Chen Q | Association between adult weight gain and colorectal cancer: a dose-response meta-analysis of observational studies. | Int J Cancer. 2015 Jun 15;136(12):2880-9. |
| Li B | Folate intake and breast cancer prognosis: a meta-analysis of prospective observational studies. | Eur J Cancer Prev. 2015 Mar;24(2):113-21. |
| Chen J | Tea and coffee consumption and risk of laryngeal cancer: a systematic review meta-analysis. | PLoS One. 2014 Dec 12;9(12):e112006. |
| Chen GC | N-3 polyunsaturated fatty acids intake and risk of colorectal cancer: meta-analysis of prospective studies. | Cancer Causes Control. 2015 Jan;26(1):133-41. |
| Liang S | Height and kidney cancer risk: a meta-analysis of prospective studies. | J Cancer Res Clin Oncol. 2015 Oct;141(10):1799-807. |
| Yang B | Biospecimen long-chain N-3 PUFA and risk of colorectal cancer: a meta-analysis of data from 60,627 individuals. | PLoS One. 2014 Nov 6;9(11):e110574. |
| Keum N | Calcium intake and colorectal cancer risk: dose-response meta-analysis of prospective observational studies. | Int J Cancer. 2014 Oct 15;135(8):1940-8. |
| Feng Y | Maternal parity and the risk of congenital heart defects in offspring: a dose-response meta-analysis of epidemiological observational studies. | PLoS One. 2014 Oct 8;9(10):e108944. |
| Wang F | Body mass index and risk of renal cell cancer: a dose-response meta-analysis of published cohort studies. | Int J Cancer. 2014 Oct 1;135(7):1673-86. |
| Xie F | Coffee consumption and risk of gastric cancer: a large updated meta-analysis of prospective studies. | Nutrients. 2014 Sep 18;6(9):3734-46. |
| Li P | Association between dietary antioxidant vitamins intake/blood level and risk of gastric cancer. | Int J Cancer. 2014 Sep 15;135(6):1444-53. |
| Abete I | Association between total, processed, red and white meat consumption and all-cause, CVD and IHD mortality: a meta-analysis of cohort studies. | Br J Nutr. 2014 Sep 14;112(5):762-75. |
| Hu MB | Obesity affects the biopsy-mediated detection of prostate cancer, particularly high-grade prostate cancer: a dose-response meta-analysis of 29,464 patients. | PLoS One. 2014 Sep 3;9(9):e106677. |
| Greenwood DC | Caffeine intake during pregnancy and adverse birth outcomes: a systematic review and dose-response meta-analysis. | Eur J Epidemiol. 2014 Oct;29(10):725-34. |
| Cai S | Alcohol drinking and the risk of colorectal cancer death: a meta-analysis. | Eur J Cancer Prev. 2014 Nov;23(6):532-9. |
| Ben Q | Alcohol drinking and the risk of colorectal adenoma: a dose-response meta-analysis. | Eur J Cancer Prev. 2015 Jul;24(4):286-95. |
| Lu D | Vitamin D status and risk of non-Hodgkin lymphoma: a meta-analysis. | Cancer Causes Control. 2014 Nov;25(11):1553-63. |
| Wang X | Fruit and vegetable consumption and mortality from all causes, cardiovascular disease, and cancer: systematic review and dose-response meta-analysis of prospective cohort studies. | BMJ. 2014 Jul 29;349:g4490. |
| Huang Y | A meta-analysis of parental smoking and the risk of childhood brain tumors. | PLoS One. 2014 Jul 24;9(7):e102910. |
| Zhou D | Nut consumption in relation to cardiovascular disease risk and type 2 diabetes: a systematic review and meta-analysis of prospective studies. | Am J Clin Nutr. 2014 Jul;100(1):270-7. |
| Zhang C | Alcohol intake and risk of stroke: a dose-response meta-analysis of prospective studies. | Int J Cardiol. 2014 Jul 1;174(3):669-77. |
| Zhang Z | Quantitative analysis of dietary protein intake and stroke risk. | Neurology. 2014 Jul 1;83(1):19-25. |
| Xue XJ | Red and processed meat consumption and the risk of lung cancer: a dose-response meta-analysis of 33 published studies. | Int J Clin Exp Med. 2014 Jun 15;7(6):1542-53. |
| Zhang YF | Folate intake and the risk of breast cancer: a dose-response meta-analysis of prospective studies. | PLoS One. 2014 Jun 16;9(6):e100044. |
| Zhang YF | Polyunsaturated fatty acid intake and risk of lung cancer: a meta-analysis of prospective studies. | PLoS One. 2014 Jun 12;9(6):e99637. |
| Lee DR | Coffee consumption and risk of fractures: a systematic review and dose-response meta-analysis. | Bone. 2014 Jun;63:20-8. |
| Kim Y | Vitamin D intake, blood 25(OH)D levels, and breast cancer risk or mortality: a meta-analysis. | Br J Cancer. 2014 May 27;110(11):2772-84. |
| Wu H | Association between dietary carbohydrate intake and dietary glycemic index and risk of age-related cataract: a meta-analysis. | Invest Ophthalmol Vis Sci. 2014 May 15;55(6):3660-8. |
| Masson P | Chronic kidney disease and the risk of stroke: a systematic review and meta-analysis. | Nephrol Dial Transplant. 2015 Jul;30(7):1162-9. |
| Wang C | Effect of drinking on all-cause mortality in women compared with men: a meta-analysis. | J Womens Health (Larchmt). 2014 May;23(5):373-81. |
| Yang WS | Tea consumption and risk of type 2 diabetes: a dose-response meta-analysis of cohort studies. | Br J Nutr. 2014 Apr 28;111(8):1329-39. |
| Zhang YF | Association between folate intake and the risk of lung cancer: a dose-response meta-analysis of prospective studies. | PLoS One. 2014 Apr 8;9(4):e93465. |
| Li J | Dietary mushroom intake may reduce the risk of breast cancer: evidence from a meta-analysis of observational studies. | PLoS One. 2014 Apr 1;9(4):e93437. |
| Li Y | Alcohol drinking and upper aerodigestive tract cancer mortality: a systematic review and meta-analysis. | Oral Oncol. 2014 Apr;50(4):269-75. |
| Cui R | Blood α-tocopherol, γ-tocopherol levels and risk of prostate cancer: a meta-analysis of prospective studies. | PLoS One. 2014 Mar 25;9(3):e93044. |
| Guan HB | Parity and pancreatic cancer risk: a dose-response meta-analysis of epidemiologic studies. | PLoS One. 2014 Mar 21;9(3):e92738. |
| Yu F | Tea consumption and the risk of five major cancers: a dose-response meta-analysis of prospective studies. | BMC Cancer. 2014 Mar 17;14:197. |
| Liu Z | Complex association between alanine aminotransferase activity and mortality in general population: a systematic review and meta-analysis of prospective studies. | PLoS One. 2014 Mar 14;9(3):e91410. |
| Di Giuseppe D | Cigarette smoking and risk of rheumatoid arthritis: a dose-response meta-analysis. | Arthritis Res Ther. 2014 Mar 5;16(2):R61. |
| Discacciati A | Coffee consumption and risk of nonaggressive, aggressive and fatal prostate cancer--a dose-response meta-analysis. | Ann Oncol. 2014 Mar;25(3):584-91. |
| Aune D | Body mass index and the risk of gout: a systematic review and dose-response meta-analysis of prospective studies. | Eur J Nutr. 2014 Dec;53(8):1591-601. |
| Xiaohua Y | The non-linear threshold association between aspirin use and esophageal adenocarcinoma: results of a dose-response meta-analysis. | Pharmacoepidemiol Drug Saf. 2014 Mar;23(3):278-84. |
| Yang W | Is heme iron intake associated with risk of coronary heart disease? A meta-analysis of prospective studies. | Eur J Nutr. 2014;53(2):395-400. |
| Zhong S | Coffee consumption and risk of prostate cancer: an up-to-date meta-analysis. | Eur J Clin Nutr. 2014 Mar;68(3):330-7. |
| Xu X | Dietary carrot consumption and the risk of prostate cancer. | Eur J Nutr. 2014 Dec;53(8):1615-23. |
| Jiang X | Coffee and caffeine intake and incidence of type 2 diabetes mellitus: a meta-analysis of prospective studies. | Eur J Nutr. 2014 Feb;53(1):25-38. |
| Cheng J | Maternal coffee consumption during pregnancy and risk of childhood acute leukemia: a metaanalysis. | Am J Obstet Gynecol. 2014 Feb;210(2):151.e1-151.e10. |
| Chen GC | Leptin levels and risk of type 2 diabetes: gender-specific meta-analysis. | Obes Rev. 2014 Feb;15(2):134-42. |
| Liu YJ | Dietary flavonoids intake and risk of type 2 diabetes: a meta-analysis of prospective cohort studies. | Clin Nutr. 2014 Feb;33(1):59-63. |
| Hu MB | Obesity has multifaceted impact on biochemical recurrence of prostate cancer: a dose-response meta-analysis of 36,927 patients. | Med Oncol. 2014 Feb;31(2):829. |
| Ding M | Caffeinated and decaffeinated coffee consumption and risk of type 2 diabetes: a systematic review and a dose-response meta-analysis. | Diabetes Care. 2014 Feb;37(2):569-86. |
| Larsson SC | Red meat and processed meat consumption and all-cause mortality: a meta-analysis. | Am J Epidemiol. 2014 Feb 1;179(3):282-9. |
| Liu XM | Dietary total flavonoids intake and risk of mortality from all causes and cardiovascular disease in the general population: A systematic review and meta-analysis of cohort studies. | Mol Nutr Food Res. 2017 Jun;61(6). |
| Lu L | Aspirin as a potential modality for the chemoprevention of breast cancer: A dose-response meta-analysis of cohort studies from 857,831 participants. | Oncotarget. 2017 Jun 20;8(25):40389-40401. |
| Turati F | Alcohol and liver cancer: a systematic review and meta-analysis of prospective studies. | Ann Oncol. 2014 Aug;25(8):1526-35. |
| Lu W | Dairy products intake and cancer mortality risk: a meta-analysis of 11 population-based cohort studies. | Nutr J. 2016 Oct 21;15(1):91. |
| Wu AM | The relationship between vitamin A and risk of fracture: meta-analysis of prospective studies. | J Bone Miner Res. 2014 Sep;29(9):2032-9. |
| Lv QB | The serum 25-hydroxyvitamin D levels and hip fracture risk: a meta-analysis of prospective cohort studies. | Oncotarget. 2017 Jun 13;8(24):39849-39858. |
| Li JW | Serum phosphate concentration and incidence of stroke: a systemic review and meta-analysis. | Neurol Sci. 2014 Dec;35(12):1877-82. |
| Mohr SB | Meta-analysis of vitamin D sufficiency for improving survival of patients with breast cancer. | Anticancer Res. 2014 Mar;34(3):1163-6. |
| Ma HQ | Effects of Serum Triglycerides on Prostate Cancer and Breast Cancer Risk: A Meta-Analysis of Prospective Studies. | Nutr Cancer. 2016 Oct;68(7):1073-82. |
| Ma L | Nut consumption and the risk of coronary artery disease: a dose-response meta-analysis of 13 prospective studies. | Thromb Res. 2014 Oct;134(4):790-4. |
| Lamm SH | A Systematic Review and Meta-Regression Analysis of Lung Cancer Risk and Inorganic Arsenic in Drinking Water. | Int J Environ Res Public Health. 2015 Dec 7;12(12):15498-515. |
| Zhu B | Allium vegetables and garlic supplements do not reduce risk of colorectal cancer, based on meta-analysis of prospective studies. | Clin Gastroenterol Hepatol. 2014 Dec;12(12):1991-2001.e1-4; quiz e121. |
| Ma X | Association between whole grain intake and all-cause mortality: a meta-analysis of cohort studies. | Oncotarget. 2016 Sep 20;7(38):61996-62005. |
| Macis D | Circulating adiponectin and breast cancer risk: a systematic review and meta-analysis. | Int J Epidemiol. 2014 Aug;43(4):1226-36. |
| Cheng M | Caffeine intake and atrial fibrillation incidence: dose response meta-analysis of prospective cohort studies. | Can J Cardiol. 2014 Apr;30(4):448-54. |
| Mao QQ | Dietary fiber intake is inversely associated with risk of pancreatic cancer: a meta-analysis. | Asia Pac J Clin Nutr. 2017 Jan;26(1):89-96. |
| Crippa A | Coffee consumption and mortality from all causes, cardiovascular disease, and cancer: a dose-response meta-analysis. | Am J Epidemiol. 2014 Oct 15;180(8):763-75. |
| Wang S | A meta-analysis of coffee intake and risk of urolithiasis. | Urol Int. 2014;93(2):220-8. |
| Huang C | Sugar sweetened beverages consumption and risk of coronary heart disease: a meta-analysis of prospective studies. | Atherosclerosis. 2014 May;234(1):11-6. |
| Mocellin S | Vitamin B6 and Cancer Risk: A Field Synopsis and Meta-Analysis. | J Natl Cancer Inst. 2017 Mar 1;109(3):1-9. |
| Huang TB | Coffee consumption and urologic cancer risk: a meta-analysis of cohort studies. | Int Urol Nephrol. 2014 Aug;46(8):1481-93. |
| Arnold LW | The HbA1c and all-cause mortality relationship in patients with type 2 diabetes is J-shaped: a meta-analysis of observational studies. | Rev Diabet Stud. 2014 Summer;11(2):138-52. |
| Mostofsky E | Alcohol and Immediate Risk of Cardiovascular Events: A Systematic Review and Dose-Response Meta-Analysis. | Circulation. 2016 Mar 8;133(10):979-87. |
| He Q | Waist circumference and risk of lower urinary tract symptoms: a meta-analysis. | Aging Male. 2014 Dec;17(4):223-9. |
| Kim Y | Dietary fiber intake and total mortality: a meta-analysis of prospective cohort studies. | Am J Epidemiol. 2014 Sep 15;180(6):565-73. |
| Neilson HK | Moderate-vigorous recreational physical activity and breast cancer risk, stratified by menopause status: a systematic review and meta-analysis. | Menopause. 2017 Mar;24(3):322-344. |
| Farvid MS | Dietary linoleic acid and risk of coronary heart disease: a systematic review and meta-analysis of prospective cohort studies. | Circulation. 2014 Oct 28;130(18):1568-78. |
| Kunutsor SK | Gamma-glutamyl transferase and risk of type II diabetes: an updated systematic review and dose-response meta-analysis. | Ann Epidemiol. 2014 Nov;24(11):809-16. |
| Ornello R | Migraine and body mass index categories: a systematic review and meta-analysis of observational studies. | J Headache Pain. 2015 Mar 28;16:27. |
| Sheng J | Coffee, tea, and the risk of hip fracture: a meta-analysis. | Osteoporos Int. 2014 Jan;25(1):141-50. |
| Sun K | Passive smoke exposure and risk of diabetes: a meta-analysis of prospective studies. | Endocrine. 2014 Nov;47(2):421-7. |
| Yao B | Dietary fiber intake and risk of type 2 diabetes: a dose-response analysis of prospective studies. | Eur J Epidemiol. 2014 Feb;29(2):79-88. |
| Guan HB | Parity and risk of colorectal cancer: a dose-response meta-analysis of prospective studies. | PLoS One. 2013 Sep 30;8(9):e75279. |
| Maalmi H | Serum 25-hydroxyvitamin D levels and survival in colorectal and breast cancer patients: systematic review and meta-analysis of prospective cohort studies. | Eur J Cancer. 2014 May;50(8):1510-21. |
| Qin W | A U-shaped association of body mass index and all-cause mortality in heart failure patients: A dose-response meta-analysis of prospective cohort studies. | Cardiovasc Ther. 2017 Apr;35(2). |
| Aune D | Maternal body mass index and the risk of fetal death, stillbirth, and infant death: a systematic review and meta-analysis. | JAMA. 2014 Apr 16;311(15):1536-46. |
| Aune D | Breastfeeding and the maternal risk of type 2 diabetes: a systematic review and dose-response meta-analysis of cohort studies. | Nutr Metab Cardiovasc Dis. 2014 Feb;24(2):107-15. |
| Zhong S | Association between physical activity and mortality in breast cancer: a meta-analysis of cohort studies. | Eur J Epidemiol. 2014 Jun;29(6):391-404. |
| Kunutsor SK | Liver enzymes and risk of all-cause mortality in general populations: a systematic review and meta-analysis. | Int J Epidemiol. 2014 Feb;43(1):187-201. |
| Qin Z | Fibroblast growth factor 23 as a predictor of cardiovascular and all-cause mortality in prospective studies. | Atherosclerosis. 2017 Jun;261:1-11. |
| Syngelaki A | Diet and exercise for preeclampsia prevention in overweight and obese pregnant women: systematic review and meta-analysis. | J Matern Fetal Neonatal Med. 2018 Sep 6:1-7. |
| Yang Y | Increased intake of vegetables, but not fruit, reduces risk for hepatocellular carcinoma: a meta-analysis. | Gastroenterology. 2014 Nov;147(5):1031-42. |
| Ran HQ | Coffee Consumption and Pancreatic Cancer Risk: An Update Meta-analysis of Cohort Studies. | Pak J Med Sci. 2016 Jan-Feb;32(1):253-9. |
| Li M | Fruit and vegetable intake and risk of type 2 diabetes mellitus: meta-analysis of prospective cohort studies. | BMJ Open. 2014 Nov 5;4(11):e005497. |
| Li B | Intake of vegetables and fruit and risk of esophageal adenocarcinoma: a meta-analysis of observational studies. | Eur J Nutr. 2014 Oct;53(7):1511-21. |
| Rong K | Increasing Level of Leisure Physical Activity Could Reduce the Risk of Hip Fracture in Older Women: A Dose-Response Meta-analysis of Prospective Cohort Studies. | Medicine (Baltimore). 2016 Mar;95(11):e2984. |
| Ma L | A dose-response meta-analysis of dietary lutein and zeaxanthin intake in relation to risk of age-related cataract. | Graefes Arch Clin Exp Ophthalmol. 2014 Jan;252(1):63-70. |
| Schlesinger S | Asymmetric and Symmetric Dimethylarginine as Risk Markers for Total Mortality and Cardiovascular Outcomes: A Systematic Review and Meta-Analysis of Prospective Studies. | PLoS One. 2016 Nov 3;11(11):e0165811. |
| Wang ZM | Flavonol intake and stroke risk: a meta-analysis of cohort studies. | Nutrition. 2014 May;30(5):518-23. |
| Qi H | Dose-response meta-analysis on coffee, tea and caffeine consumption with risk of Parkinson's disease. | Geriatr Gerontol Int. 2014 Apr;14(2):430-9. |
| Schwingshackl L | Food groups and risk of type 2 diabetes mellitus: a systematic review and meta-analysis of prospective studies. | Eur J Epidemiol. 2017 May;32(5):363-375. |
| Wang Q | Consumption of fruit, but not vegetables, may reduce risk of gastric cancer: results from a meta-analysis of cohort studies. | Eur J Cancer. 2014 May;50(8):1498-509. |
| Schwingshackl L | Food groups and risk of all-cause mortality: a systematic review and meta-analysis of prospective studies. | Am J Clin Nutr. 2017 Jun;105(6):1462-1473. |
| Dibaba DT | Dietary magnesium intake and risk of metabolic syndrome: a meta-analysis. | Diabet Med. 2014 Nov;31(11):1301-9. |
| Smith AD | Physical activity and incident type 2 diabetes mellitus: a systematic review and dose-response meta-analysis of prospective cohort studies. | Diabetologia. 2016 Dec;59(12):2527-2545. |
| Song H | Maternal vitamin D status during pregnancy and risk of childhood asthma: A meta-analysis of prospective studies. | Mol Nutr Food Res. 2017 May;61(5). |
| Yao B | Intake of fruit and vegetables and risk of bladder cancer: a dose-response meta-analysis of observational studies. | Cancer Causes Control. 2014 Dec;25(12):1645-58. |
| Farrokhi F | Association between depression and mortality in patients receiving long-term dialysis: a systematic review and meta-analysis. | Am J Kidney Dis. 2014 Apr;63(4):623-35. |
| Cao S | Association of quantity and duration of smoking with erectile dysfunction: a dose-response meta-analysis. | J Sex Med. 2014 Oct;11(10):2376-84. |
| Tamez M | Egg consumption and risk of incident type 2 diabetes: a dose-response meta-analysis of prospective cohort studies. | Br J Nutr. 2016 Jun;115(12):2212-8. |
| Tang Z | Dietary flavonoid intake and the risk of stroke: a dose-response meta-analysis of prospective cohort studies. | BMJ Open. 2016 Jun 8;6(6):e008680. |
| Yu XF | Fish consumption and risk of gastrointestinal cancers: a meta-analysis of cohort studies. | World J Gastroenterol. 2014 Nov 7;20(41):15398-412. |
| Tajima R | High cholesterol intake is associated with elevated risk of type 2 diabetes mellitus - a meta-analysis. | Clin Nutr. 2014 Dec;33(6):946-50. |
| Zhou ZY | Body mass index and knee osteoarthritis risk: a dose-response meta-analysis. | Obesity (Silver Spring). 2014 Oct;22(10):2180-5. |
| Jiang W | Dairy foods intake and risk of Parkinson's disease: a dose-response meta-analysis of prospective cohort studies. | Eur J Epidemiol. 2014 Sep;29(9):613-9. |
| Tong X | Cheese Consumption and Risk of All-Cause Mortality: A Meta-Analysis of Prospective Studies. | Nutrients. 2017 Jan 13;9(1). pii: E63. |
| Wu ZJ | C-reactive protein and risk of fracture: a systematic review and dose-response meta-analysis of prospective cohort studies. | Osteoporos Int. 2015 Jan;26(1):49-57. |
| Shi Y | Dose-response meta-analysis of poultry intake and colorectal cancer incidence and mortality. | Eur J Nutr. 2015 Mar;54(2):243-50. |
| Threapleton DE | Dietary fibre intake and risk of cardiovascular disease: systematic review and meta-analysis. | BMJ. 2013 Dec 19;347:f6879. |
| Zhou ZY | Dietary methionine intake and risk of incident colorectal cancer: a meta-analysis of 8 prospective studies involving 431,029 participants. | PLoS One. 2013 Dec 10;8(12):e83588. |
| Liu Z | Elevated alanine aminotransferase is strongly associated with incident metabolic syndrome: a meta-analysis of prospective studies. | PLoS One. 2013 Dec 4;8(12):e80596. |
| Guan HB | Parity and kidney cancer risk: evidence from epidemiologic studies. | Cancer Epidemiol Biomarkers Prev. 2013 Dec;22(12):2345-53. |
| Verbeek J | Occupational Exposure to Knee Loading and the Risk of Osteoarthritis of the Knee: A Systematic Review and a Dose-Response Meta-Analysis. | Saf Health Work. 2017 Jun;8(2):130-142. |
| Vieira AR | Fruits, vegetables and lung cancer risk: a systematic review and meta-analysis. | Ann Oncol. 2016 Jan;27(1):81-96. |
| Wang D | Serum 25-hydroxyvitamin D and breast cancer risk: a meta-analysis of prospective studies. | Tumour Biol. 2013 Dec;34(6):3509-17. |
| Qu X | Consumption of red and processed meat and risk for esophageal squamous cell carcinoma based on a meta-analysis. | Ann Epidemiol. 2013 Dec;23(12):762-770.e1. |
| Vinceti M | Meta-Analysis of Potassium Intake and the Risk of Stroke. | J Am Heart Assoc. 2016 Oct 6;5(10). pii: e004210. |
| Ding M | Long-term coffee consumption and risk of cardiovascular disease: a systematic review and a dose-response meta-analysis of prospective cohort studies. | Circulation. 2014 Feb 11;129(6):643-59. |
| Macacu A | Active and passive smoking and risk of breast cancer: a meta-analysis. | Breast Cancer Res Treat. 2015 Nov;154(2):213-24. |
| Aune D | Whole grain and refined grain consumption and the risk of type 2 diabetes: a systematic review and dose-response meta-analysis of cohort studies. | Eur J Epidemiol. 2013 Nov;28(11):845-58. |
| Xu X | Dietary intake of vitamins A, C, and E and the risk of colorectal adenoma: a meta-analysis of observational studies. | Eur J Cancer Prev. 2013 Nov;22(6):529-39. |
| Wang J | Coffee consumption and the risk of cutaneous melanoma: a meta-analysis. | Eur J Nutr. 2016 Jun;55(4):1317-29. |
| Wang J | Alcohol consumption and risk of periodontitis: a meta-analysis. | J Clin Periodontol. 2016 Jul;43(7):572-83. |
| Wang J | Gamma-glutamyltransferase and risk of cardiovascular mortality: A dose-response meta-analysis of prospective cohort studies. | PLoS One. 2017 Feb 23;12(2):e0172631. |
| Luan NN | Breastfeeding and ovarian cancer risk: a meta-analysis of epidemiologic studies. | Am J Clin Nutr. 2013 Oct;98(4):1020-31. |
| Aune D | Dairy products and the risk of type 2 diabetes: a systematic review and dose-response meta-analysis of cohort studies. | Am J Clin Nutr. 2013 Oct;98(4):1066-83. |
| Wang K | Smoking increases risks of all-cause and breast cancer specific mortality in breast cancer individuals: a dose-response meta-analysis of prospective cohort studies involving 39725 breast cancer cases. | Oncotarget. 2016 Dec 13;7(50):83134-83147. |
| Wu W | Association of vitamin B6, vitamin B12 and methionine with risk of breast cancer: a dose-response meta-analysis. | Br J Cancer. 2013 Oct 1;109(7):1926-44. |
| Wang L | Coffee and caffeine consumption and depression: A meta-analysis of observational studies. | Aust N Z J Psychiatry. 2016 Mar;50(3):228-42. |
| Wang Q | Parity and osteoporotic fracture risk in postmenopausal women: a dose-response meta-analysis of prospective studies. | Osteoporos Int. 2016 Jan;27(1):319-30. |
| Wang W | Association of dairy products consumption with risk of obesity in children and adults: a meta-analysis of mainly cross-sectional studies. | Ann Epidemiol. 2016 Dec;26(12):870-882.e2. |
| Gao D | Dairy products consumption and risk of type 2 diabetes: systematic review and dose-response meta-analysis. | PLoS One. 2013 Sep 27;8(9):e73965. |
| Wang X | Red and processed meat consumption and mortality: dose-response meta-analysis of prospective cohort studies. | Public Health Nutr. 2016 Apr;19(5):893-905. |
| Wang XL | Association between serum selenium level and type 2 diabetes mellitus: a non-linear dose-response meta-analysis of observational studies. | Nutr J. 2016 May 4;15(1):48. |
| Wang YT | Association between alcohol intake and the risk of pancreatic cancer: a dose-response meta-analysis of cohort studies. | BMC Cancer. 2016 Mar 12;16:212. |
| Xu W | Alcohol consumption and dementia risk: a dose-response meta-analysis of prospective studies. | Eur J Epidemiol. 2017 Jan;32(1):31-42. |
| Li Y | Egg consumption and risk of cardiovascular diseases and diabetes: a meta-analysis. | Atherosclerosis. 2013 Aug;229(2):524-30. |
| Xue WQ | Quantitative association of tobacco smoking with the risk of nasopharyngeal carcinoma: a comprehensive meta-analysis of studies conducted between 1979 and 2011. | Am J Epidemiol. 2013 Aug 1;178(3):325-38. |
| Weng H | Tea Consumption and Risk of Bladder Cancer: A Dose-Response Meta-Analysis. | Front Physiol. 2017 Jan 23;7:693. |
| Chen Y | Body mass index and risk of gastric cancer: a meta-analysis of a population with more than ten million from 24 prospective studies. | Cancer Epidemiol Biomarkers Prev. 2013 Aug;22(8):1395-408. |
| Wu J | Dietary Protein Sources and Incidence of Breast Cancer: A Dose-Response Meta-Analysis of Prospective Studies. | Nutrients. 2016 Nov 17;8(11). pii: E730. |
| Amadou A | Overweight, obesity and risk of premenopausal breast cancer according to ethnicity: a systematic review and dose-response meta-analysis. | Obes Rev. 2013 Aug;14(8):665-78. |
| Ye X | Frequency-risk and duration-risk relationships between aspirin use and gastric cancer: a systematic review and meta-analysis. | PLoS One. 2013 Jul 30;8(7):e71522. |
| Wu L | Sleep duration and falls: a systemic review and meta-analysis of observational studies. | J Sleep Res. 2017 Jun;26(3):293-301. |
| Liu J | Intake of fruit and vegetables and risk of esophageal squamous cell carcinoma: a meta-analysis of observational studies. | Int J Cancer. 2013 Jul 15;133(2):473-85. |
| Wu L | Coffee intake and the incident risk of cognitive disorders: A dose-response meta-analysis of nine prospective cohort studies. | Clin Nutr. 2017 Jun;36(3):730-736. |
| Wu L | Fruit and vegetables consumption and incident hypertension: dose-response meta-analysis of prospective cohort studies. | J Hum Hypertens. 2016 Oct;30(10):573-80. |
| Lin HL | Folate intake and pancreatic cancer risk: an overall and dose-response meta-analysis. | Public Health. 2013 Jul;127(7):607-13. |
| Qin J | Oral contraceptive use and uterine leiomyoma risk: a meta-analysis based on cohort and case-control studies. | Arch Gynecol Obstet. 2013 Jul;288(1):139-48. |
| Chen GC | Fruits and vegetables consumption and risk of non-Hodgkin's lymphoma: a meta-analysis of observational studies. | Int J Cancer. 2013 Jul;133(1):190-200. |
| Malerba S | A meta-analysis of prospective studies of coffee consumption and mortality for all causes, cancers and cardiovascular diseases. | Eur J Epidemiol. 2013 Jul;28(7):527-39. |
| Kim DH | Adiponectin levels and the risk of hypertension: a systematic review and meta-analysis. | Hypertension. 2013 Jul;62(1):27-32. |
| Wang X | Cholesterol levels and risk of hemorrhagic stroke: a systematic review and meta-analysis. | Stroke. 2013 Jul;44(7):1833-9. |
| Ben Q | Association Between Consumption of Fruits and Vegetables and Risk of Colorectal Adenoma: A PRISMA-Compliant Meta-Analysis of Observational Studies. | Medicine (Baltimore). 2015 Oct;94(42):e1599. |
| Qiao L | Intakes of heme iron and zinc and colorectal cancer incidence: a meta-analysis of prospective studies. | Cancer Causes Control. 2013 Jun;24(6):1175-83. |
| Xu T | Statin Adherence and the Risk of Stroke: A Dose-Response Meta-Analysis. | CNS Drugs. 2017 Apr;31(4):263-271. |
| Jiang W | Coffee and caffeine intake and breast cancer risk: an updated dose-response meta-analysis of 37 published studies. | Gynecol Oncol. 2013 Jun;129(3):620-9. |
| Xu W | Education and Risk of Dementia: Dose-Response Meta-Analysis of Prospective Cohort Studies. | Mol Neurobiol. 2016 Jul;53(5):3113-3123. |
| Salehi M | Meat, fish, and esophageal cancer risk: a systematic review and dose-response meta-analysis. | Nutr Rev. 2013 May;71(5):257-67. |
| Ju SY | Sleep duration and metabolic syndrome in adult populations: a meta-analysis of observational studies. | Nutr Diabetes. 2013 May 13;3:e65. |
| Yang C | Red Meat Consumption and the Risk of Stroke: A Dose-Response Meta-analysis of Prospective Cohort Studies. | J Stroke Cerebrovasc Dis. 2016 May;25(5):1177-1186. |
| Larsson SC | Dietary calcium intake and risk of stroke: a dose-response meta-analysis. | Am J Clin Nutr. 2013 May;97(5):951-7. |
| Yang Y | Alcohol consumption and risk of coronary artery disease: A dose-response meta-analysis of prospective studies. | Nutrition. 2016 Jun;32(6):637-44. |
| Song Y | Blood 25-hydroxy vitamin D levels and incident type 2 diabetes: a meta-analysis of prospective studies. | Diabetes Care. 2013 May;36(5):1422-8. |
| Bauer SR | Plasma vitamin D levels, menopause, and risk of breast cancer: dose-response meta-analysis of prospective studies. | Medicine (Baltimore). 2013 May;92(3):123-31. |
| Yi C | Statins intake and risk of liver cancer: A dose-response meta analysis of prospective cohort studies. | Medicine (Baltimore). 2017 Jul;96(27):e7435. |
| Yi X | Breastfeeding and thyroid cancer risk in women: A dose-response meta-analysis of epidemiological studies. | Clin Nutr. 2016 Oct;35(5):1039-46. |
| Xu B | No evidence of decreased risk of colorectal adenomas with white meat, poultry, and fish intake: a meta-analysis of observational studies. | Ann Epidemiol. 2013 Apr;23(4):215-22. |
| Yu L | Dietary vitamin B2 intake and breast cancer risk: a systematic review and meta-analysis. | Arch Gynecol Obstet. 2017 Mar;295(3):721-729. |
| Yuan S | Chocolate Consumption and Risk of Coronary Heart Disease, Stroke, and Diabetes: A Meta-Analysis of Prospective Studies. | Nutrients. 2017 Jul 2;9(7). pii: E688. |
| Zeng L | Macronutrient Intake and Risk of Crohn's Disease: Systematic Review and Dose-Response Meta-Analysis of Epidemiological Studies. | Nutrients. 2017 May 15;9(5). pii: E500. |
| Zeng Y | Parity and All-cause Mortality in Women and Men: A Dose-Response Meta-Analysis of Cohort Studies. | Sci Rep. 2016 Jan 13;6:19351. |
| Qu X | Magnesium and the risk of cardiovascular events: a meta-analysis of prospective cohort studies. | PLoS One. 2013;8(3):e57720. |
| Li YH | Fish consumption and incidence of heart failure: a meta-analysis of prospective cohort studies. | Chin Med J (Engl). 2013 Mar;126(5):942-8. |
| Zhang D | Is aspirin use associated with a decreased risk of ovarian cancer? A systematic review and meta-analysis of observational studies with dose-response analysis. | Gynecol Oncol. 2016 Aug;142(2):368-77. |
| Zheng JS | Effects of green tea, black tea, and coffee consumption on the risk of esophageal cancer: a systematic review and meta-analysis of observational studies. | Nutr Cancer. 2013;65(1):1-16. |
| Livesey G | Is there a dose-response relation of dietary glycemic load to risk of type 2 diabetes? Meta-analysis of prospective cohort studies. | Am J Clin Nutr. 2013 Mar;97(3):584-96. |
| Jin M | Alcohol drinking and all cancer mortality: a meta-analysis. | Ann Oncol. 2013 Mar;24(3):807-16. |
| Zhang D | Can Aspirin Reduce the Risk of Endometrial Cancer?: A Systematic Review and Meta-analysis of Observational Studies. | Int J Gynecol Cancer. 2016 Jul;26(6):1111-20. |
| Kunutsor SK | Vitamin D and risk of future hypertension: meta-analysis of 283,537 participants. | Eur J Epidemiol. 2013 Mar;28(3):205-21. |
| Qu X | Nonlinear association between magnesium intake and the risk of colorectal cancer. | Eur J Gastroenterol Hepatol. 2013 Mar;25(3):309-18. |
| Ye X | Dose-risk and duration-risk relationships between aspirin and colorectal cancer: a meta-analysis of published cohort studies. | PLoS One. 2013;8(2):e57578. |
| Wang Y | Effect of Carotene and Lycopene on the Risk of Prostate Cancer: A Systematic Review and Dose-Response Meta-Analysis of Observational Studies. | PLoS One. 2015 Sep 15;10(9):e0137427. |
| Wu Y | Physical activity and risk of breast cancer: a meta-analysis of prospective studies. | Breast Cancer Res Treat. 2013 Feb;137(3):869-82. |
| Zhang D | Is maternal smoking during pregnancy associated with an increased risk of congenital heart defects among offspring? A systematic review and meta-analysis of observational studies. | J Matern Fetal Neonatal Med. 2017 Mar;30(6):645-657. |
| Zhang R | Serum 25-hydroxyvitamin D and the risk of cardiovascular disease: dose-response meta-analysis of prospective studies. | Am J Clin Nutr. 2017 Apr;105(4):810-819. |
| Xu X | Red and processed meat intake and risk of colorectal adenomas: a meta-analysis of observational studies. | Int J Cancer. 2013 Jan 15;132(2):437-48. |
| Rong Y | Egg consumption and risk of coronary heart disease and stroke: dose-response meta-analysis of prospective cohort studies. | BMJ. 2013 Jan 7;346:e8539. |
| Wang X | Inflammatory markers and risk of type 2 diabetes: a systematic review and meta-analysis. | Diabetes Care. 2013 Jan;36(1):166-75. |
| Jia Z | Serum uric acid levels and incidence of impaired fasting glucose and type 2 diabetes mellitus: a meta-analysis of cohort studies. | Diabetes Res Clin Pract. 2013 Jul;101(1):88-96. |
| Zhang X | Selenium status and cardiovascular diseases: meta-analysis of prospective observational studies and randomized controlled trials. | Eur J Clin Nutr. 2016 Feb;70(2):162-9. |
| Zhang Y | Intakes of fish and polyunsaturated fatty acids and mild-to-severe cognitive impairment risks: a dose-response meta-analysis of 21 cohort studies. | Am J Clin Nutr. 2016 Feb;103(2):330-40. |
| Zhao J | Dietary fat intake and endometrial cancer risk: A dose response meta-analysis. | Medicine (Baltimore). 2016 Jul;95(27):e4121. |
| Zhao L | Association of body mass index with bladder cancer risk: a dose-response meta-analysis of prospective cohort studies. | Oncotarget. 2017 May 16;8(20):33990-34000. |
| Fan J | Dietary glycemic index, glycemic load, and risk of coronary heart disease, stroke, and stroke mortality: a systematic review with meta-analysis. | PLoS One. 2012;7(12):e52182. |
| Zhao LG | Fish consumption and all-cause mortality: a meta-analysis of cohort studies. | Eur J Clin Nutr. 2016 Feb;70(2):155-61. |
| Ju SY | Serum 25-hydroxyvitamin D levels and the risk of depression: a systematic review and meta-analysis. | J Nutr Health Aging. 2013;17(5):447-55. |
| Zhao R | Steroid therapy and the risk of osteonecrosis in SARS patients: a dose-response meta-analysis. | Osteoporos Int. 2017 Mar;28(3):1027-1034. |
| Yu XF | A meta-analysis of the effects of energy intake on risk of digestive cancers. | World J Gastroenterol. 2012 Dec 28;18(48):7362-70. |
| Larsson SC | Long-chain omega-3 polyunsaturated fatty acids and risk of stroke: a meta-analysis. | Eur J Epidemiol. 2012 Dec;27(12):895-901. |
| Zhao Y | Folate intake, serum folate levels and esophageal cancer risk: an overall and dose-response meta-analysis. | Oncotarget. 2017 Feb 7;8(6):10458-10469. |
| Zhong C | Sex-Specific Relationship Between Serum Uric Acid and Risk of Stroke: A Dose-Response Meta-Analysis of Prospective Studies. | J Am Heart Assoc. 2017 Mar 29;6(4). pii: e005042. |
| Zhong CK | Measures of Abdominal Adiposity and Risk of Stroke: A Dose-Response Meta-analysis of Prospective Studies. | Biomed Environ Sci. 2016 Jan;29(1):12-23. |
| Zhong GC | HbA1c and Risks of All-Cause and Cause-Specific Death in Subjects without Known Diabetes: A Dose-Response Meta-Analysis of Prospective Cohort Studies. | Sci Rep. 2016 Apr 5;6:24071. |
| Chen GC | Magnesium intake and risk of colorectal cancer: a meta-analysis of prospective studies. | Eur J Clin Nutr. 2012 Nov;66(11):1182-6. |
| Hong S | Abdominal obesity and the risk of colorectal adenoma: a meta-analysis of observational studies. | Eur J Cancer Prev. 2012 Nov;21(6):523-31. |
| Wang Y | Body mass index and risk of primary liver cancer: a meta-analysis of prospective studies. | Oncologist. 2012;17(11):1461-8. |
| Zhong S | Body mass index and mortality in prostate cancer patients: a dose-response meta-analysis. | Prostate Cancer Prostatic Dis. 2016 Jun;19(2):122-31. |
| Zhong S | β-Blocker use and mortality in cancer patients: systematic review and meta-analysis of observational studies. | Eur J Cancer Prev. 2016 Sep;25(5):440-8. |
| Kaluza J | Red meat consumption and risk of stroke: a meta-analysis of prospective studies. | Stroke. 2012 Oct;43(10):2556-60. |
| Zhou Q | Does alcohol consumption modify the risk of endometrial cancer? A dose-response meta-analysis of prospective studies. | Arch Gynecol Obstet. 2017 Feb;295(2):467-479. |
| Zhu YJ | Association of dietary vitamin E intake with risk of lung cancer: a dose-response meta-analysis. | Asia Pac J Clin Nutr. 2017 Mar;26(2):271-277. |
| Je Y | Coffee consumption and risk of endometrial cancer: findings from a large up-to-date meta-analysis. | Int J Cancer. 2012 Oct 1;131(7):1700-10. |
| Rui R | Excess body mass index and risk of liver cancer: a nonlinear dose-response meta-analysis of prospective studies. | PLoS One. 2012;7(9):e44522. |
| Bellocco R | Alcohol drinking and risk of renal cell carcinoma: results of a meta-analysis. | Ann Oncol. 2012 Sep;23(9):2235-44. |
| Wang S | Body mass index and risk of BPH: a meta-analysis. | Prostate Cancer Prostatic Dis. 2012 Sep;15(3):265-72. |
| Zhou Y | Association of fish and n-3 fatty acid intake with the risk of type 2 diabetes: a meta-analysis of prospective studies. | Br J Nutr. 2012 Aug;108(3):408-17. |
| Ma XY | Glycemic load, glycemic index and risk of cardiovascular diseases: meta-analyses of prospective studies. | Atherosclerosis. 2012 Aug;223(2):491-6. |
| Aune D | Height and pancreatic cancer risk: a systematic review and meta-analysis of cohort studies. | Cancer Causes Control. 2012 Aug;23(8):1213-22. |
| Aune D | Dietary compared with blood concentrations of carotenoids and breast cancer risk: a systematic review and meta-analysis of prospective studies. | Am J Clin Nutr. 2012 Aug;96(2):356-73. |
| Shen L | Tea consumption and risk of stroke: a dose-response meta-analysis of prospective studies. | J Zhejiang Univ Sci B. 2012 Aug;13(8):652-62. |
| Mostofsky E | Habitual coffee consumption and risk of heart failure: a dose-response meta-analysis. | Circ Heart Fail. 2012 Jul 1;5(4):401-5. |
| Discacciati A | Body mass index and incidence of localized and advanced prostate cancer--a dose-response meta-analysis of prospective studies. | Ann Oncol. 2012 Jul;23(7):1665-71. |
| Zhou Y | A dose-response meta-analysis of coffee consumption and bladder cancer. | Prev Med. 2012 Jul;55(1):14-22. |
| Aune D | Dietary fiber and breast cancer risk: a systematic review and meta-analysis of prospective studies. | Ann Oncol. 2012 Jun;23(6):1394-402. |
| Dahabreh IJ | Parity and risk of lung cancer in women: systematic review and meta-analysis of epidemiological studies. | Lung Cancer. 2012 May;76(2):150-8. |
| Wang ZM | Flavonols intake and the risk of coronary heart disease: a meta-analysis of cohort studies. | Atherosclerosis. 2012 May;222(1):270-3. |
| Jiang L | Body mass index and susceptibility to knee osteoarthritis: a systematic review and meta-analysis. | Joint Bone Spine. 2012 May;79(3):291-7. |
| Hu EA | White rice consumption and risk of type 2 diabetes: meta-analysis and systematic review. | BMJ. 2012 Mar 15;344:e1454. |
| Aune D | Body mass index, abdominal fatness and pancreatic cancer risk: a systematic review and non-linear dose-response meta-analysis of prospective studies. | Ann Oncol. 2012 Apr;23(4):843-52. |
| Wallin A | Fish consumption, dietary long-chain n-3 fatty acids, and risk of type 2 diabetes: systematic review and meta-analysis of prospective studies. | Diabetes Care. 2012 Apr;35(4):918-29. |
| Pan A | α-Linolenic acid and risk of cardiovascular disease: a systematic review and meta-analysis. | Am J Clin Nutr. 2012 Dec;96(6):1262-73. |
| Ward AM | Home measurement of blood pressure and cardiovascular disease: systematic review and meta-analysis of prospective studies. | J Hypertens. 2012 Mar;30(3):449-56. |
| Larsson SC | Dietary magnesium intake and risk of stroke: a meta-analysis of prospective studies. | Am J Clin Nutr. 2012 Feb;95(2):362-6. |
| Larsson SC | Red and processed meat consumption and risk of pancreatic cancer: meta-analysis of prospective studies. | Br J Cancer. 2012 Jan 31;106(3):603-7. |
| Liao WC | Blood glucose concentration and risk of pancreatic cancer: systematic review and dose-response meta-analysis. | BMJ. 2015 Jan 2;350:g7371. |
| Chung M | Vitamin D with or without calcium supplementation for prevention of cancer and fractures: an updated meta-analysis for the U.S. Preventive Services Task Force. | Ann Intern Med. 2011 Dec 20;155(12):827-38. |
| Bagnardi V | Alcohol consumption and lung cancer risk in never smokers: a meta-analysis. | Ann Oncol. 2011 Dec;22(12):2631-9. |
| Larsson SC | Fish consumption and the risk of stroke: a dose-response meta-analysis. | Stroke. 2011 Dec;42(12):3621-3. |
| Larsson SC | Coffee consumption and risk of stroke: a dose-response meta-analysis of prospective studies. | Am J Epidemiol. 2011 Nov 1;174(9):993-1001. |
| Larsson SC | Body mass index and risk of non-Hodgkin's and Hodgkin's lymphoma: a meta-analysis of prospective studies. | Eur J Cancer. 2011 Nov;47(16):2422-30. |
| Larsson SC | Dietary potassium intake and risk of stroke: a dose-response meta-analysis of prospective studies. | Stroke. 2011 Oct;42(10):2746-50. |
| Samitz G | Domains of physical activity and all-cause mortality: systematic review and dose-response meta-analysis of cohort studies. | Int J Epidemiol. 2011 Oct;40(5):1382-400. |
| Fedirko V | Alcohol drinking and colorectal cancer risk: an overall and dose-response meta-analysis of published studies. | Ann Oncol. 2011 Sep;22(9):1958-72. |
| Burgers AM | Meta-analysis and dose-response metaregression: circulating insulin-like growth factor I (IGF-I) and mortality. | J Clin Endocrinol Metab. 2011 Sep;96(9):2912-20. |
| Tong X | Dairy consumption and risk of type 2 diabetes mellitus: a meta-analysis of cohort studies. | Eur J Clin Nutr. 2011 Sep;65(9):1027-31. |
| Dong JY | Dietary fiber intake and risk of breast cancer: a meta-analysis of prospective cohort studies. | Am J Clin Nutr. 2011 Sep;94(3):900-5. |
| Pilz S | Vitamin D status and mortality risk in CKD: a meta-analysis of prospective studies. | Am J Kidney Dis. 2011 Sep;58(3):374-82. |
| Xu XT | Meta-analysis: circulating adiponectin levels and risk of colorectal cancer and adenoma. | J Dig Dis. 2011 Aug;12(4):234-44. |
| Cheng G | Alcohol intake and risk of renal cell carcinoma: a meta-analysis of published case-control studies. | Arch Med Sci. 2011 Aug;7(4):648-57. |
| Alexander DD | Meta-analysis of prospective studies of red meat consumption and colorectal cancer. | Eur J Cancer Prev. 2011 Jul;20(4):293-307. |
| Zhou Y | Consumption of large amounts of Allium vegetables reduces risk for gastric cancer in a meta-analysis. | Gastroenterology. 2011 Jul;141(1):80-9. |
| Zhang Z | Habitual coffee consumption and risk of hypertension: a systematic review and meta-analysis of prospective observational studies. | Am J Clin Nutr. 2011 Jun;93(6):1212-9. |
| Bischoff-Ferrari HA | Milk intake and risk of hip fracture in men and women: a meta-analysis of prospective cohort studies. | J Bone Miner Res. 2011 Apr;26(4):833-9. |
| Burgaz A | Blood 25-hydroxyvitamin D concentration and hypertension: a meta-analysis. | J Hypertens. 2011 Apr;29(4):636-45. |
| Wallin A | Red and processed meat consumption and risk of ovarian cancer: a dose-response meta-analysis of prospective studies. | Br J Cancer. 2011 Mar 29;104(7):1196-201. |
| Gandini S | Meta-analysis of observational studies of serum 25-hydroxyvitamin D levels and colorectal, breast and prostate cancer and colorectal adenoma. | Int J Cancer. 2011 Mar 15;128(6):1414-24. |
| Yu X | Coffee consumption and risk of cancers: a meta-analysis of cohort studies. | BMC Cancer. 2011 Mar 15;11:96. |
| Dong J | Coffee drinking and pancreatic cancer risk: a meta-analysis of cohort studies. | World J Gastroenterol. 2011 Mar 7;17(9):1204-10. |
| Woodcock J | Non-vigorous physical activity and all-cause mortality: systematic review and meta-analysis of cohort studies. | Int J Epidemiol. 2011 Feb;40(1):121-38. |
| Dong JY | Soy isoflavones consumption and risk of breast cancer incidence or recurrence: a meta-analysis of prospective studies. | Breast Cancer Res Treat. 2011 Jan;125(2):315-23. |
| Soedamah-Muthu SS | Milk and dairy consumption and incidence of cardiovascular diseases and all-cause mortality: dose-response meta-analysis of prospective cohort studies. | Am J Clin Nutr. 2011 Jan;93(1):158-71. |
| Heine-Bröring RC | Dietary supplement use and colorectal cancer risk: a systematic review and meta-analyses of prospective cohort studies. | Int J Cancer. 2015 May 15;136(10):2388-401. |
| Keum N | Leisure-time physical activity and endometrial cancer risk: dose-response meta-analysis of epidemiological studies. | Int J Cancer. 2014 Aug 1;135(3):682-94. |
| Kunutsor SK | Gamma glutamyltransferase, alanine aminotransferase and risk of cancer: systematic review and meta-analysis. | Int J Cancer. 2015 Mar 1;136(5):1162-70. |
| Liu CF | Gamma-glutamyltransferase levels and risk of metabolic syndrome: a meta-analysis of prospective cohort studies. | Int J Clin Pract. 2012 Jul;66(7):692-8. |
| Roerecke M | The cardioprotective association of average alcohol consumption and ischaemic heart disease: a systematic review and meta-analysis. | Addiction. 2012 Jul;107(7):1246-60. |
| Kim Y | Dairy consumption and risk of metabolic syndrome: a meta-analysis. | Diabet Med. 2016 Apr;33(4):428-40. |
| Patra J | Dose-response relationship between alcohol consumption before and during pregnancy and the risks of low birthweight, preterm birth and small for gestational age (SGA)-a systematic review and meta-analyses. | BJOG. 2011 Nov;118(12):1411-21. |
| Feng LP | Breastfeeding and the risk of ovarian cancer: a meta-analysis. | J Midwifery Womens Health. 2014 Jul-Aug;59(4):428-37. |
| Liu L | Fiber consumption and all-cause, cardiovascular, and cancer mortalities: a systematic review and meta-analysis of cohort studies. | Mol Nutr Food Res. 2015 Jan;59(1):139-46. |
| Aune D | Anthropometric factors and ovarian cancer risk: a systematic review and nonlinear dose-response meta-analysis of prospective studies. | Int J Cancer. 2015 Apr 15;136(8):1888-98. |
| Kodama S | Quantitative relationship between body weight gain in adulthood and incident type 2 diabetes: a meta-analysis. | Obes Rev. 2014 Mar;15(3):202-14. |
| Gong TT | Circulating adiponectin, leptin and adiponectin-leptin ratio and endometrial cancer risk: Evidence from a meta-analysis of epidemiologic studies. | Int J Cancer. 2015 Oct 15;137(8):1967-78. |
| Rota M | Alcohol drinking and cutaneous melanoma risk: a systematic review and dose-risk meta-analysis. | Br J Dermatol. 2014 May;170(5):1021-8. |
| Jarl J | Time pattern of reduction in risk of oesophageal cancer following alcohol cessation--a meta-analysis. | Addiction. 2012 Jul;107(7):1234-43. |
| Tamariz L | Uric acid as a predictor of all-cause mortality in heart failure: a meta-analysis. | Congest Heart Fail. 2011 Jan-Feb;17(1):25-30. |
| Guha N | Betel quid chewing and the risk of oral and oropharyngeal cancers: a meta-analysis with implications for cancer control. | Int J Cancer. 2014 Sep 15;135(6):1433-43. |
| Zheng H | Body mass index and risk of knee osteoarthritis: systematic review and meta-analysis of prospective studies. | BMJ Open. 2015 Dec 11;5(12):e007568. |
| Chan DS | Circulating C-Reactive Protein and Breast Cancer Risk-Systematic Literature Review and Meta-analysis of Prospective Cohort Studies. | Cancer Epidemiol Biomarkers Prev. 2015 Oct;24(10):1439-49. |
| Chen GC | Dairy products consumption and metabolic syndrome in adults: systematic review and meta-analysis of observational studies. | Sci Rep. 2015 Sep 29;5:14606. |
| Liu Z | Dose-response Relationship of Serum Uric Acid with Metabolic Syndrome and Non-alcoholic Fatty Liver Disease Incidence: A Meta-analysis of Prospective Studies. | Sci Rep. 2015 Sep 23;5:14325. |
| Sun JW | Association Between Television Viewing Time and All-Cause Mortality: A Meta-Analysis of Cohort Studies. | Am J Epidemiol. 2015 Dec 1;182(11):908-16. |
| Duan P | Body mass index and risk of lung cancer: Systematic review and dose-response meta-analysis. | Sci Rep. 2015 Nov 19;5:16938. |
| Jiang L | Dietary fat intake and endometrial cancer risk: dose-response meta-analysis of epidemiological studies. | Sci Rep. 2015 Nov 16;5:16693. |
| Yang L | Red and Processed Meat Consumption Increases Risk for Non-Hodgkin Lymphoma: A PRISMA-Compliant Meta-Analysis of Observational Studies. | Medicine (Baltimore). 2015 Nov;94(45):e1729. |
| Yuan H | Serum Uric Acid Levels and Risk of Metabolic Syndrome: A Dose-Response Meta-Analysis of Prospective Studies. | J Clin Endocrinol Metab. 2015 Nov;100(11):4198-207. |
| Kunutsor SK | Gamma-glutamyltransferase and risk of hypertension: a systematic review and dose-response meta-analysis of prospective evidence. | J Hypertens. 2015 Dec;33(12):2373-81. |
| Ruan H | Habitual Sleep Duration and Risk of Childhood Obesity: Systematic Review and Dose-response Meta-analysis of Prospective Cohort Studies. | Sci Rep. 2015 Nov 5;5:16160. |
| Lv QB | The relationship between weight change and risk of hip fracture: meta-analysis of prospective studies. | Sci Rep. 2015 Nov 2;5:16030. |
| Shi Y | Household physical activity and cancer risk: a systematic review and dose-response meta-analysis of epidemiological studies. | Sci Rep. 2015 Oct 7;5:14901. |
| Larsson SC | Milk Consumption and Mortality from All Causes, Cardiovascular Disease, and Cancer: A Systematic Review and Meta-Analysis. | Nutrients. 2015 Sep 11;7(9):7749-63. |
| Zhou Q | Coffee consumption and risk of endometrial cancer: a dose-response meta-analysis of prospective cohort studies. | Sci Rep. 2015 Aug 25;5:13410. |
| Lv H | Parity and Cardiovascular Disease Mortality: a Dose-Response Meta-Analysis of Cohort Studies. | Sci Rep. 2015 Aug 24;5:13411. |
| Knott C | Alcohol Consumption and the Risk of Type 2 Diabetes: A Systematic Review and Dose-Response Meta-analysis of More Than 1.9 Million Individuals From 38 Observational Studies. | Diabetes Care. 2015 Sep;38(9):1804-12. |
| Aune D | Anthropometric factors and endometrial cancer risk: a systematic review and dose-response meta-analysis of prospective studies. | Ann Oncol. 2015 Aug;26(8):1635-48. |
| Lim JE | Body concentrations of persistent organic pollutants and prostate cancer: a meta-analysis. | Environ Sci Pollut Res Int. 2015 Aug;22(15):11275-84. |
| Aune D | Resting heart rate and the risk of type 2 diabetes: A systematic review and dose--response meta-analysis of cohort studies. | Nutr Metab Cardiovasc Dis. 2015 Jun;25(6):526-34. |
| Chen F | Association of vitamin C, vitamin D, vitamin E and risk of bladder cancer: a dose-response meta-analysis. | Sci Rep. 2015 Apr 23;5:9599. |
| Wu AM | The relationship between dietary protein consumption and risk of fracture: a subgroup and dose-response meta-analysis of prospective cohort studies. | Sci Rep. 2015 Mar 16;5:9151. |
| Chen LW | Maternal caffeine intake during pregnancy is associated with risk of low birth weight: a systematic review and dose-response meta-analysis. | BMC Med. 2014 Sep 19;12:174. |
| Chen GC | N-3 polyunsaturated fatty acids intake and risk of colorectal cancer: meta-analysis of prospective studies. | Cancer Causes Control. 2015 Jan;26(1):133-41. |
| Ju SY | Dietary magnesium intake and metabolic syndrome in the adult population: dose-response meta-analysis and meta-regression. | Nutrients. 2014 Dec 22;6(12):6005-19. |
| Xia X | Body mass index and risk of breast cancer: a nonlinear dose-response meta-analysis of prospective studies. | Sci Rep. 2014 Dec 15;4:7480. |
| Chen P | Lycopene and Risk of Prostate Cancer: A Systematic Review and Meta-Analysis. | Medicine (Baltimore). 2015 Aug;94(33):e1260. |
| Kelly P | Systematic review and meta-analysis of reduction in all-cause mortality from walking and cycling and shape of dose response relationship. | Int J Behav Nutr Phys Act. 2014 Oct 24;11:132. |
| Luo J | Association between vitamin C intake and lung cancer: a dose-response meta-analysis. | Sci Rep. 2014 Aug 22;4:6161. |
| Wu W | Coffee consumption and bladder cancer: a meta-analysis of observational studies. | Sci Rep. 2015 Mar 12;5:9051. |
| Di Giuseppe D | Fish consumption and risk of rheumatoid arthritis: a dose-response meta-analysis. | Arthritis Res Ther. 2014 Sep 30;16(5):446. |
| Fu YQ | Effect of individual omega-3 fatty acids on the risk of prostate cancer: a systematic review and dose-response meta-analysis of prospective cohort studies. | J Epidemiol. 2015;25(4):261-74. |
| Goodman M | Dose-response relationship between serum 2,3,7,8-tetrachlorodibenzo-p-dioxin and diabetes mellitus: a meta-analysis. | Am J Epidemiol. 2015 Mar 15;181(6):374-84. |
| Greenwood DC | Glycemic index, glycemic load, carbohydrates, and type 2 diabetes: systematic review and dose-response meta-analysis of prospective studies. | Diabetes Care. 2013 Dec;36(12):4166-71. |
| Park M | Body mass index and biliary tract disease: a systematic review and meta-analysis of prospective studies. | Prev Med. 2014 Aug;65:13-22. |
| Shan Z | Sleep duration and risk of type 2 diabetes: a meta-analysis of prospective studies. | Diabetes Care. 2015 Mar;38(3):529-37. |
| Tian DY | Calcium intake and the risk of stroke: an up-dated meta-analysis of prospective studies. | Asia Pac J Clin Nutr. 2015;24(2):245-52. |
| Xu C | Fruits and vegetables intake and risk of bladder cancer: a PRISMA-compliant systematic review and dose-response meta-analysis of prospective cohort studies. | Medicine (Baltimore). 2015 May;94(17):e759. |
| Wang X | Dietary calcium intake and mortality risk from cardiovascular disease and all causes: a meta-analysis of prospective cohort studies. | BMC Med. 2014 Sep 25;12:158. |
| Keum N | Adult weight gain and adiposity-related cancers: a dose-response meta-analysis of prospective observational studies. | J Natl Cancer Inst. 2015 Mar 10;107(2). pii: djv088. |
| Aune D | Anthropometric factors and ovarian cancer risk: a systematic review and nonlinear dose-response meta-analysis of prospective studies. | Int J Cancer. 2015 Apr 15;136(8):1888-98. |
| Wang B | Effect of long-term exposure to air pollution on type 2 diabetes mellitus risk: a systemic review and meta-analysis of cohort studies. | Eur J Endocrinol. 2014 Nov;171(5):R173-82. |
| Chen B | Tea consumption didn't modify the risk of fracture: a dose-response meta-analysis of observational studies. | Diagn Pathol. 2014 Mar 3;9:44. |
| Liu M | Lack of effects of dietary folate intake on risk of breast cancer: an updated meta-analysis of prospective studies. | Asian Pac J Cancer Prev. 2014;15(5):2323-8. |
| Jin Z | Alcohol consumption as a preventive factor for developing rheumatoid arthritis: a dose-response meta-analysis of prospective studies. | Ann Rheum Dis. 2014 Nov;73(11):1962-7. |
| Leung Yinko SS | Fish consumption and acute coronary syndrome: a meta-analysis. | Am J Med. 2014 Sep;127(9):848-57.e2. |
| Greenwood DC | Association between sugar-sweetened and artificially sweetened soft drinks and type 2 diabetes: systematic review and dose-response meta-analysis of prospective studies. | Br J Nutr. 2014 Sep 14;112(5):725-34. |
| Ju SY | Blood vitamin D status and metabolic syndrome in the general adult population: a dose-response meta-analysis. | J Clin Endocrinol Metab. 2014 Mar;99(3):1053-63. |
| Zou L | Non-linear dose-response relationship between cigarette smoking and pancreatic cancer risk: evidence from a meta-analysis of 42 observational studies. | Eur J Cancer. 2014 Jan;50(1):193-203. |
| Zhao H | Sleep duration and cancer risk: a systematic review and meta-analysis of prospective studies. | Asian Pac J Cancer Prev. 2013;14(12):7509-15. |
| Chen GC | Vitamin C intake, circulating vitamin C and risk of stroke: a meta-analysis of prospective studies. | J Am Heart Assoc.2013 Nov 27;2(6):e000329. |
| Wang F | A meta-analysis on dose-response relationship between night shift work and the risk of breast cancer. | Ann Oncol. 2013 Nov;24(11):2724-32. |
| Ijaz S | Night-shift work and breast cancer--a systematic review and meta-analysis. | Scand J Work Environ Health. 2013 Sep 1;39(5):431-47. |
| Del Gobbo LC | Circulating and dietary magnesium and risk of cardiovascular disease: a systematic review and meta-analysis of prospective studies. | Am J Clin Nutr. 2013 Jul;98(1):160-73. |
| Kanhai DA | Adiponectin and incident coronary heart disease and stroke. A systematic review and meta-analysis of prospective studies. | Obes Rev. 2013 Jul;14(7):555-67. |
| Nie ZL | Magnesium intake and incidence of stroke: meta-analysis of cohort studies. | Nutr Metab Cardiovasc Dis. 2013 Mar;23(3):169-76. |
| Xie Q | Isoflavone consumption and risk of breast cancer: a dose-response meta-analysis of observational studies. | Asia Pac J Clin Nutr. 2013;22(1):118-27. |
| Fry JS | Systematic review with meta-analysis of the epidemiological evidence relating FEV1 decline to lung cancer risk. | BMC Cancer. 2012 Oct 27;12:498. |
| Wang L | Circulating 25-hydroxy-vitamin D and risk of cardiovascular disease: a meta-analysis of prospective studies. | Circ Cardiovasc Qual Outcomes. 2012 Nov;5(6):819-29. |
| Soedamah-Muthu SS | Dairy consumption and incidence of hypertension: a dose-response meta-analysis of prospective cohort studies. | Hypertension. 2012 Nov;60(5):1131-7. |
| Aune D | Dietary fructose, carbohydrates, glycemic indices and pancreatic cancer risk: a systematic review and meta-analysis of cohort studies. | Ann Oncol. 2012 Oct;23(10):2536-46. |
| Druesne-Pecollo N | Excess body weight and second primary cancer risk after breast cancer: a systematic review and meta-analysis of prospective studies. | Breast Cancer Res Treat. 2012 Oct;135(3):647-54. |
| Wang ZM | Folate and risk of coronary heart disease: a meta-analysis of prospective studies. | Nutr Metab Cardiovasc Dis. 2012 Oct;22(10):890-9. |
| McLeod DS | Thyrotropin and thyroid cancer diagnosis: a systematic review and dose-response meta-analysis. | J Clin Endocrinol Metab. 2012 Aug;97(8):2682-92. |
| Aune D | Fruits, vegetables and breast cancer risk: a systematic review and meta-analysis of prospective studies. | Breast Cancer Res Treat. 2012 Jul;134(2):479-93. |
| Hurst R | Selenium and prostate cancer: systematic review and meta-analysis. | Am J Clin Nutr. 2012 Jul;96(1):111-22. |
| Aune D | Carbohydrates, glycemic index, glycemic load, and colorectal cancer risk: a systematic review and meta-analysis of cohort studies. | Cancer Causes Control. 2012 Apr;23(4):521-35. |
| Aune D | Dairy products and colorectal cancer risk: a systematic review and meta-analysis of cohort studies. | Ann Oncol. 2012 Jan;23(1):37-45. |
| Aune D | Dietary fibre, whole grains, and risk of colorectal cancer: systematic review and dose-response meta-analysis of prospective studies. | BMJ. 2011 Nov 10;343:d6617. |
| Aune D | Nonlinear reduction in risk for colorectal cancer by fruit and vegetable intake based on meta-analysis of prospective studies. | Gastroenterology. 2011 Jul;141(1):106-18. |
| Chan DS | Red and processed meat and colorectal cancer incidence: meta-analysis of prospective studies. | PLoS One. 2011;6(6):e20456. |
| Touvier M | Meta-analyses of vitamin D intake, 25-hydroxyvitamin D status, vitamin D receptor polymorphisms, and colorectal cancer risk. | Cancer Epidemiol Biomarkers Prev. 2011 May;20(5):1003-16. |
| Gilbert R | Associations of circulating and dietary vitamin D with prostate cancer risk: a systematic review and dose-response meta-analysis. | Cancer Causes Control. 2011 Mar;22(3):319-40. |
| Wang R | Folate intake, serum folate levels, and prostate cancer risk: a meta-analysis of prospective studies. | BMC Public Health. 2014 Dec 29;14:1326. |
| Zheng JS | Intake of fish and marine n-3 polyunsaturated fatty acids and risk of breast cancer: meta-analysis of data from 21 independent prospective cohort studies. | BMJ. 2013 Jun 27;346:f3706. |
| Afshin A | Consumption of nuts and legumes and risk of incident ischemic heart disease, stroke, and diabetes: a systematic review and meta-analysis. | Am J Clin Nutr. 2014 Jul;100(1):278-88. |
